# The reference genome of *Miscanthus floridulus* illuminates the evolution of Saccharinae

**DOI:** 10.1038/s41477-021-00908-y

**Published:** 2021-05-06

**Authors:** Guobin Zhang, Chunxia Ge, Pingping Xu, Shukai Wang, Senan Cheng, Yanbin Han, Yancui Wang, Yongbin Zhuang, Xinwei Hou, Ting Yu, Xitong Xu, Shuhan Deng, Quanquan Li, Yinqing Yang, Xiaoru Yin, Weidong Wang, Wenxue Liu, Chunxiao Zheng, Xuezhen Sun, Zhenlin Wang, Ray Ming, Shuting Dong, Jianxin Ma, Xiansheng Zhang, Cuixia Chen

**Affiliations:** 1 State Key Laboratory of Crop Biology, Shandong Agricultural University, Taian, China; 2 College of Agronomy, Shandong Agricultural University, Taian, China; 3 Novogene Bioinformatics Institute, Beijing, China; 4 Department of Plant Biology, University of Illinois at Urbana-Champaign, Urbana, IL USA; 5 Department of Agronomy, Purdue University, West Lafayette, IN USA

**Keywords:** Genomics, Biofuels, Plant evolution

## Abstract

*Miscanthus*, a member of the Saccharinae subtribe that includes sorghum and sugarcane, has been widely studied as a feedstock for cellulosic biofuel production. Here, we report the sequencing and assembly of the *Miscanthus floridulus* genome by the integration of PacBio sequencing and Hi-C mapping, resulting in a chromosome-scale, high-quality reference genome of the genus *Miscanthus*. Comparisons among Saccharinae genomes suggest that *Sorghum* split first from the common ancestor of *Saccharum* and *Miscanthus*, which subsequently diverged from each other, with two successive whole-genome duplication events occurring independently in the *Saccharum* genus and one whole-genome duplication occurring in the *Miscanthus* genus. Fusion of two chromosomes occurred during rediploidization in *M. floridulus* and no significant subgenome dominance was observed. A survey of cellulose synthases (CesA) in *M. floridulus* revealed quite high expression of most *CesA* genes in growing stems, which is in agreement with the high cellulose content of this species. Resequencing and comparisons of 75 *Miscanthus* accessions suggest that *M. lutarioriparius* is genetically close to *M. sacchariflorus* and that *M. floridulus* is more distantly related to other species and is more genetically diverse. This study provides a valuable genomic resource for molecular breeding and improvement of *Miscanthus* and Saccharinae crops.

## Abstract (web summary)

*Miscanthus* is widely used as a feedstock for cellulosic biofuel production. Genome sequencing shows that *M. floridulus* is most distantly related to other *Miscanthus* species and is more genetically diverse.

## Main

The Saccharinae subtribe is within Andropogoneae tribe in the grass family (Poaceae). It is comprised of the *Saccharum* complex, the interspecific breeding group inclusive of *Saccharum* (sugarcane) and *Miscanthus*, and the Sorghinae subtribe, which has been merged into subtribe Saccharinae recently^1^. In this group, *Miscanthus*, *Sorghum* and *Saccharum* are widely studied for their favourable characteristics for bioenergy production^2–5^. In addition to their capability for C_4_ photosynthesis, they require low nitrogen for growth and can regrow after harvest. In sorghum, photosynthetically fixed carbon is mainly stored in the form of starch in grain and cellulose/hemicellulose in leaves and stems. Sugarcane stores most fixed carbon as free sugars in stems and cellulose/hemicellulose in stems and leaves, while abundant cellulose/hemicellulose as the main form of fixed carbon is predominantly stored in both stems and leaves of *Miscanthus*. Briefly, *Miscanthus* can yield ~30 t ha^−1^ dry biomass, comprising 41–46% cellulose and 29% hemicellulose on average^6,7^ and sorghum can produce ~15–25 t ha^−1^ dry biomass, comprising 26–29% cellulose and 20–26% hemicellulose on average^8,9^, whereas commercial sugarcane yields 39 t ha^−1^ dry biomass comprising 14–29% cellulose and 9–18% hemicellulose^5,10^. Thus, among these three species, *Miscanthus* provides the most cellulose/hemicellulose per unit area for bioenergy production. Moreover, with increasing demand for food there is a corresponding increase in the demand for agricultural land for crop production. *Miscanthus*, which can grow robustly on marginal lands such as saline or infertile lands, is considered to be the most promising energy crop species compared with sorghum and sugarcane^4,6,11^.

Polyploidization is among the strongest drivers of angiosperm evolution, contributing to the speciation and emergence of valuable traits in Saccharinae especially sugarcane and *Miscanthus*^12–14^. *Miscanthus* and sugarcane share a common ancestor, which split from the sorghum clade before their divergence. Following a single whole-genome duplication (WGD) event and chromosomal rearrangement, *Miscanthus* has a basic chromosome number of 19 (2*n* = 2*x* = 38 or 2*n* = 4*x* = 76), whereas sugarcane has a basic chromosome number of eight (*Saccharum spontaneum*) or ten (*S. officinarum*); these numbers were ultimately attained after two or more rounds of polyploidization accompanied by extensive genome rearrangement^12,15^. In general, both sugarcane and *Miscanthus* are categorized as neopolyploids, having undergone WGD within the last 5 million years^14^. Unlike in palaeopolyploids, a variety of evolutionary processes such as the activation of genes or retrotransposons, gene loss, gene silencing and subfunctionalization of genes remain ongoing^13,14^. Thus, Saccharinae may serve as a tractable system for studying potential impacts of genome duplication, subsequent subgenome divergence, genome rearrangement and extensive gene fractionation.

In monocots, members of the cellulose synthase (*CesA*) family and seven cellulose synthase-like (*Csl*) families, including *CslA*, *CslC*, *CslD*, *CslE*, *CslF*, *CslH* and *CslJ*, are generally believed to mediate the synthesis of cellulose and the hemicellulose polysaccharides respectively^16–20^. In the *CesA* lineage, *OsCesA4*, *OsCesA7* and *OsCesA9* are mainly required for cellulose synthesis in the secondary cell wall of rice, whereas *OsCesA1*, *OsCesA3* and *OsCesA8* are strongly co-expressed in tissues typical of primary cell walls^21,22^, with the function of *OsCesA5/OsCesA6* being partially redundant with that of *OsCesA3*. Because of their great importance in cell wall synthesis and bioenergy production, gene expression and phylogeny of all genes belonging to the *CesA/Csl* superfamily have been extensively studied since their identification^16–18,21–25^. In *Miscanthus*, however, only a handful of *CesA* genes were identified and analysed on the basis of transcriptome sequencing and quantitative polymerase chain reaction (qPCR), and the comprehensive genome-wide identification and expression profiling of *CesA/Csl* genes superfamily are still lacking^26,27^.

The reference genomes of sorghum and sugarcane (*S. spontaneum*) have been previously reported^15,28,29^, given their economic importance as major sources of food and livestock feed. However, despite it being one of the most promising bioenergy crops, *Miscanthus* still does not have a high-quality reference genome available. Here, we report the genome assembly of *M. floridulus*—the chromosome-scale reference genome for the genus *Miscanthus*. The assembly of the *M. floridulus* genome and comparative genomic analysis of this genome with those of sorghum and sugarcane, provide an opportunity to systematically understand the evolutionary history of the Saccharinae group, the diversification of the *Miscanthus* clade and the underlying genetic basis for the high cellulose content of *Miscanthus*.

## Results

### Genome sequencing and assembly

Due to the highly heterozygous nature of the *M. floridulus* genome, we first performed an initial genome survey to determine genome size and composition via Illumina sequencing data. The *k*-mer analysis (*k* = 17; Supplementary Fig. 1) revealed a heterozygosity rate of 1.89% and an estimated genome size of 2,462.35 megabases (Mb) (Supplementary Table 1), which was slightly smaller than the previous estimation of 2,596.59 Mb on the basis of cytometry data^30^. In addition, repeat sequences were estimated to constitute 73.42% of the genome.

We then combined four different technologies for the de novo assembly of the *M. floridulus* genome: Illumina short-read sequencing, PacBio long-read sequencing, 10x Genomics technology and Hi-C technology. After the initial assembly was achieved via FALCON with PacBio data, FALCON-Unzip was applied to the initial assembly to produce a phased diploid assembly^31^, which included primary contigs and haplotigs to address the issue of genomic heterozygosity. After phased polishing was performed, FALCON-Phase combined the primary contigs and haplotigs together with Hi-C data to extend the phasing between the unzipped haplotig blocks^32^: that is, the heterozygous regions of the genome as bubbles in the assembly graphs, resulting in the generation of two new and better phased assemblies (Supplementary Note 1). One of the phased assemblies was chosen for further polishing with PacBio reads and Illumina short reads, and yielded an assembly consisting of 4,522 contigs with an N50 length of 820,435 base pairs (bp). The phased assembly was then integrated together with the 10x Genomics data to create a hybrid assembly consisting of 2,803 scaffolds totalling 2.68 gigabases (Gb) with an N50 length of 1,644,988 bp. At the final step, a Hi-C-based physical map (Supplementary Fig. 2) was used to assemble 19 pseudochromosomes that anchored 2.44 Gb of the genome, accounting for 91.03% of the whole-genome assembly (Supplementary Table 2). A high-density genetic map comprising 3,799 single-nucleotide polymorphisms (SNPs) was used to verify the Hi-C assembly, providing evidence supporting the consistency between the two methods in terms of both chromosomal assignment and order (Supplementary Fig. 3). Additionally, the Hi-C assembly was verified by Bionano optical mapping data. The scaffolds assembled by the resulting Bionano optical maps covered 91.40% of the assembled genomes (Supplementary Table 3) and were highly consistent with the Hi-C assembly with only 186 conflicts identified between the Hi-C assembled genomes and Bionano optical maps (Supplementary Data 1). These conflicts were subsequently corrected manually (Supplementary Fig. 4).

The completeness of the assembled *M. floridulus* genome was validated using the core eukaryotic genes mapping approach (CEGMA)^33^ and benchmarking universal single-copy orthologues (BUSCO)^34^. CEGMA analysis revealed that the assembled genome covered 224 (90%) complete gene models of the 248 core eukaryotic genes (CEGs) and partially covered 14 additional CEG models (Supplementary Table 4). When BUSCO was used, among 1,440 conserved genes in our assembly, 1,378 complete single-copy plant orthologues (96%) were recalled (Supplementary Table 5). The draft assembly was further evaluated by mapping short reads to the genome assembly, which revealed a mapping rate and genome coverage of 98.4% and 99.8%, respectively (Supplementary Table 6). In addition, long terminal repeat retrotransposons assembly index (LAI), a reference-free genome metric for assessment of the assembly of repeat sequences^35^, was used to evaluate the continuity of the genome assembly. This analysis resulted in an LAI assembly score of 9.32, which is close to the quality of a reference genome according to the classification system^35^. Collectively, these results validated the high level of completeness and reliability of our *M. floridulus* genome assembly.

### Genome annotation

In total, we identified 63.6% of the genome as transposable elements (TEs), which were categorized as long terminal repeat retrotransposons (LTR-RTs) (55.8%), non-LTR-RTs (1.2%) and DNA transposons (6.6%) (Supplementary Table 7). Among the LTR-RTs, *Gypsy* elements (31.3%) were much more abundant than *Copia* elements (14.2%). In the *M. floridulus* genome, similar to those of other species, TEs tend to accumulate in intergenic regions particularly recombination-suppressed pericentromeric regions. In addition, the latest LTR-RT proliferation occurred within the last 4 million years (Supplementary Fig. 5).

**Fig. 1:**
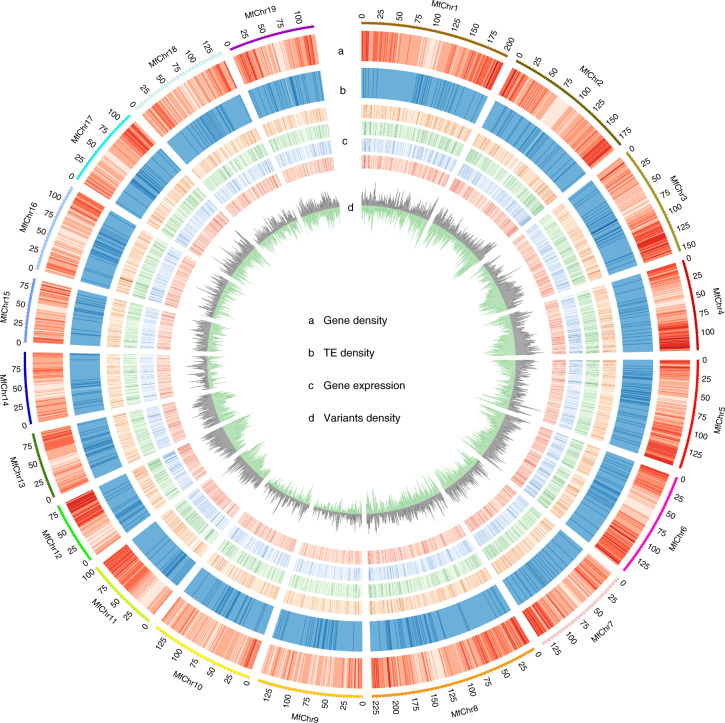
Genomic features of *M. floridulus* genome. Tracks indicate the following: gene density (a); TE density (b); gene expression level (TPM; the tracks from outermost to innermost indicate root, stem, leaf and inflorescence respectively) (c); SNP/Indels density from the resequencing data of the 75 accessions (outer track: SNP density; inner circle: Indel density) (d). Chr, chromosome. The outer circle represents the chromosome length of *M. floridulus*, with units in Mb.

A set of 76,913 genes was annotated with high confidence using a comprehensive strategy that combined de novo gene prediction, protein-based homology searches and transcriptome-based predictions, and the results showed that protein-coding genes were distributed mainly on the ends of chromosome arms (Fig. 1). Functional annotations of all predicted genes resulted in the assignment of putative functional annotations for 71,637 (93.14%) genes. Comparison among *Zea mays*, *Oryza sativa*, *Sorghum bicolor*, *S. spontaneum* and *M. floridulus* revealed that a core set of 13,539 gene families were shared among all five grass genomes and that 2,219 gene families were unique to *M. floridulus* (Supplementary Fig. 6). Gene ontology (GO) enrichment analysis showed that these *M. floridulus*-specific genes were enriched in GO categories such as postembryonic development, phosphoprotein phosphatase activity, regulation of root development, defence response to oomycetes and other basic molecular functions (Supplementary Table 8). Comparison among the three Saccharinae species revealed that they shared 18,995 gene families (Supplementary Fig. 7) and GO enrichment analysis notably revealed enrichment for the GO term ‘xyloglucan metabolic process’ (Supplementary Table 9), echoing the common view that these grasses are highly promising biofuel feedstocks^3^.

### Genome synteny and the evolutionary history of Saccharinae

**Fig. 2:**
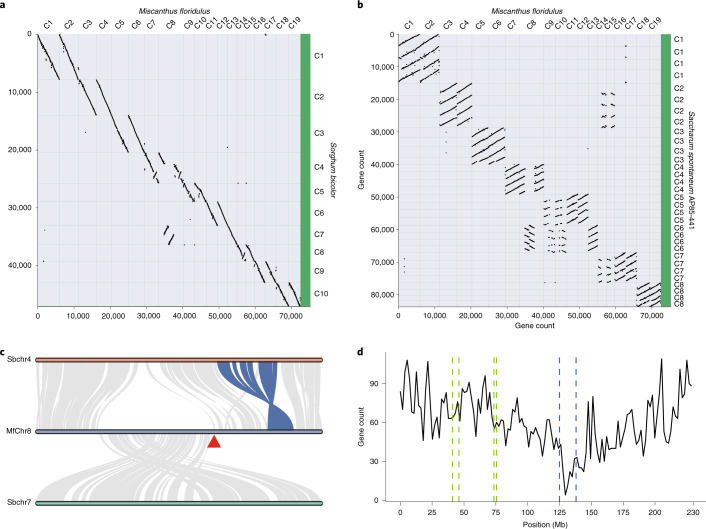
Alignment of *M. floridulus* chromosomes with sorghum or *S. spontaneum* chromosomes. **a**, A set of two homoeologous chromosomes aligned to a single sorghum chromosome except MfChr7, MfChr13 and MfChr8, among which MfChr7 aligned to Sbchr4, MfChr13 aligned to Sbchr7 and MfChr8 aligned to Sbchr4 and Sbchr7. **b**, Alignment of *M. floridulus* chromosomes with *S. spontaneum* chromosomes. **c**, The split chromosome homoeologous to Sbchr7 were inserted into the chromosome homoeologous to SbChr4 in *M. floridulus*. The red triangle indicates the centromere of MfChr8. Note that there is an inversion in Sbchr4 compared with MfChr7, MfChr8 (blue lines) and SsChr4ABCD^15^. **d**, Gene count along the MfChr8 chromosome using 2 Mb as a window and 200 kb as a shift. Dashed lines indicate the joint regions linking the split chromosome segments homoeologous to Sbchr7 and Sbchr4 and the third region (blue dashed lines) corresponds to the new evolved centromere of MfChr8. Source data

Given that the annotated genome sequences of both sorghum and sugarcane are currently available^15,28,29^, we performed genomic synteny analyses among the three species of Saccharinae. We identified 2:1 and 2:4 syntenic depth ratios for alignments between *M. floridulus*–*S. bicolor* (Fig. 2a) and *M. floridulus*–*S. spontaneum* (Fig. 2b), respectively, thereby suggesting that the ancestor of *Miscanthus* had experienced one round of WGD since its divergence from sorghum. In addition, we concurrently identified the two subgenomes of *Miscanthus* (Supplementary Fig. 8). Furthermore, as revealed by a previously constructed genetic map of *M. sinensis*^36^, the ancestor of Sbchr7 was split around the centromere region into two segments. These two segments joined together at their telomere ends and formed a new, larger segment, which was then translocated into the centromeric region of the ancestral homoeologous chromosome of Sbchr4 to form MfChr8 (Fig. 2c and Supplementary Table 10). MfChr8 presented the lowest gene density in the second region connecting the split segments of ancestors of Sbchr4 and Sbchr7, suggesting that this region was most probably the newly evolved centromere of MfChr8 (Fig. 2c and d). This fusion resulted in the reduction from two sets of ten duplicated chromosomes produced by WGD to 19 chromosomes. In addition, Sbchr4 carried an inversion (Fig. 2c) specific to the sorghum lineage that was absent in both *Miscanthus* and sugarcane, which is consistent with previous observations^15^.

**Fig. 3:**
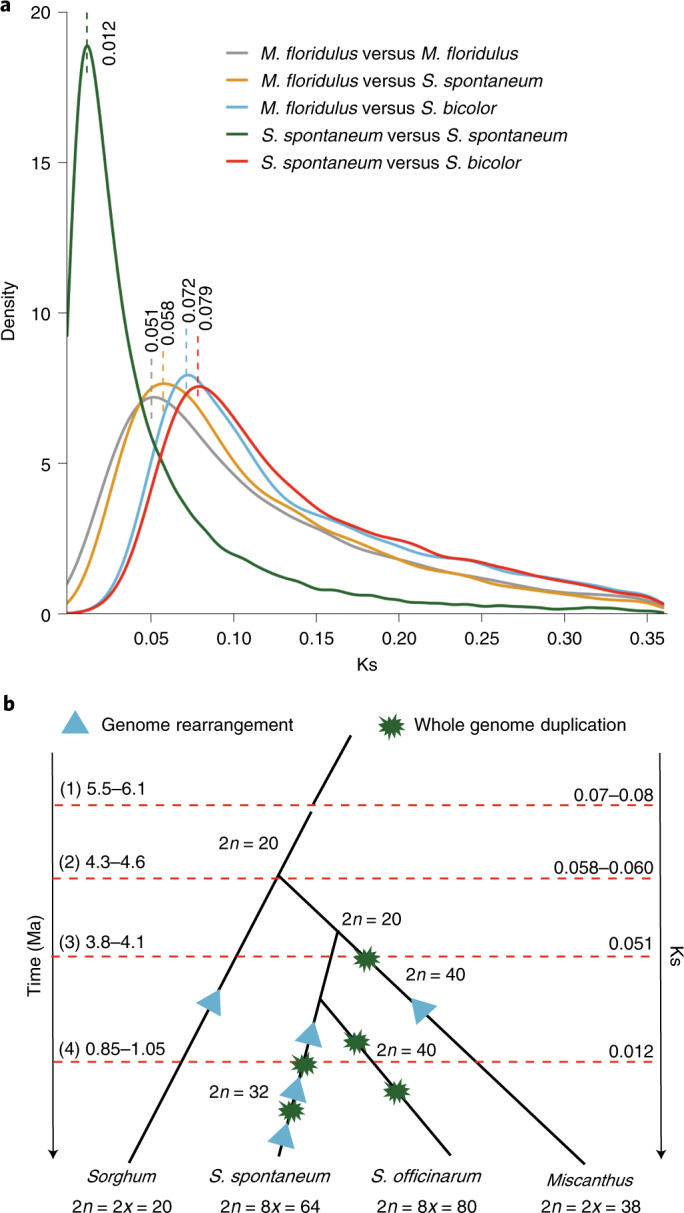
Evolutionary history of Saccharinae. **a**, Distribution of synonymous nucleotide substitutions between inter- or intraspecies. The lines with different colours represents the Ks distribution of syntelogues between two species or subgenomes of same species. **b**, A schematic species tree outlining the evolutionary history of Saccharinae group. The dark green explosion shapes show WGD events and light blue triangles indicate genome rearrangement events. A time line (leftmost) is shown in Ma and a Ks line (rightmost) is also displayed. Chromosome numbers of each state of a plant are shown along branches. (1) Time of divergence of *Miscanthus* and *Saccharum* with *Sorghum.* (2) Time of divergence of *Misanthus* with *Saccharum*. (3) Time of divergence of *Miscanthus* subgenomes. (4) Time of autopolyploidization of the ancestor of *S. spontaneum*. The plot was based on our data and analyses plus part of the results from a previous paper^15^. Source data

Previous studies have proposed that *Miscanthus* and *Saccharum* share a common allopolyploid ancestor that diverged from the *Sorghum* lineage ~3.8–4.6 million years ago (Ma)^12^. However, a recent study demonstrated that *S. spontaneum* experienced two rounds of WGD autopolyploidization, which occurred during only a brief interval of time^15^. To elucidate the genomic changes that occurred preceding and following the divergence of these three lineages, we calculated the Ks (synonymous substitutions per site) values between all homoeologous gene pairs in the intergenomic or intragenomic synteny blocks and plotted the Ks distributions of all the syntelogues. This analysis revealed that the *Saccharum*–*Sorghum* divergence (Ks = 0.079) and *Miscanthus*–*Sorghum* divergence (Ks = 0.072) occurred at approximately the same time (Fig. 3a). Although the *Miscanthus*–*Saccharum* divergence (Ks = 0.058) and the divergence of the *Miscanthus* subgenomes (Ks = 0.051) occurred at a similar time, the divergence of *Miscanthus* subgenomes occurred approximately half-a-million years later. Since the two rounds of *S. spontaneum* autopolyploidization occurred within a relatively narrow timeframe, only one Ks peak was detected to represent the two events. Compared with the Ks peak value for syntelogues of *Miscanthus*–*Saccharum*, the peak Ks value (Ks = 0.012) for syntelogues between the *S. spontaneum* subgenomes was very small, indicating that the autopolyploidization event within the *Saccharum* lineage occurred much later than the *Miscanthus*–*Saccharum* divergence.

Given that the *Miscanthus* subgenomes diverged from each other at a similar time as each of them diverged from *Saccharum*, which type of polyploidization the *Miscanthus* genome has undergone remains unclear. Previous studies suggested that the subgenomes of allopolyploids tend to exhibit subgenome dominance after genome rediploidization^13,14,37–39^. To determine if such dominance exists in *Miscanthus* and, if so, which subgenome predominated over the other, we first analysed gene losses in the synteny blocks between the two *Miscanthus* subgenomes through comparisons with their syntenic blocks in *Sorghum*. The results (Supplementary Table 11) showed that the average gene loss was ~25.8%, with no significant difference in overall gene loss between the two sets of homoeologous chromosomes (that is, two subgenomes; binomial test, *P* = 0.11) except for MfChr14-Sbchr8-MfChr15 (18.9% versus 58.3%). Notably, MfChr8, the fused chromosome derived from the ancestral Sbchr4 and Sbchr7 chromosomes, appeared to have lost fewer genes than did MfChr7 (20.9% versus 27.1%) and MfChr13 (19.2% versus 23.5%). These observations suggest that the *Miscanthus* subgenomes did not experience biased gene loss.

We then compared the gene expression levels of the syntelogues between the two subgenomes of *Miscanthus* (Supplementary Note 2) and found that there was no significant difference in the average expression level between genes in any two homoeologous chromosomes in any of the sampled tissues (Supplementary Fig. 9), including the fused chromosome MfChr8 and its non-fused chromosome homoeologues MfChr7 or MfChr13. These observations, together with unbiased gene losses from the two subgenomes suggest a lack of substantial global subgenome dominance in *Miscanthus*.

On the basis of previous reports, our own findings and the assumption that the synonymous substitution rate for coding genes is 6.5 × 10^−9^ synonymous substitutions yr^−1^ for grasses^12,15,36,40^, we were able to deduce the evolutionary history of the Saccharinae clade (Fig. 3b). The sorghum lineage diverged from the common ancestor of *Miscanthus* and *Saccharum* between 5.5 and 6.1 Ma, which then diverged from each other between 4.3 and 4.6 Ma. The ancestor of *Saccharum* then maintained a diploid state (2*n* = 20) for ~3.0 Myr, during which the ancestor of *S. spontaneum* underwent genome rearrangement and chromosome number reduction that resulted in a diploid state of 2*n* = 16. Approximately 1.0 Ma, the ancestor of *S. spontaneum* experienced the first round of autopolyploidization. Further genome rearrangement and a second round of WGD occurred sequentially and shortly after the first WGD. All these genomic changes followed by another slight genome rearrangement led to the speciation of *S. spontaneum*. Alternatively, two rounds of WGD occurred in the last 4 Myr and presumably gave rise to the basic chromosomes specific to the modern *S. officinarum*. In contrast, the ancestor of *Miscanthus* was most likely to experience an autopolyploidization event shortly after its divergence from the ancestor of *Saccharum*. When the tetraploid *Miscanthus* ancestor underwent the rediploidization process, chromosome fusion and gene losses occurred, contributing to further evolution towards the present forms of the *Miscanthus* genome.

### Cellulose synthases in *Miscanthus*

**Fig. 4:**
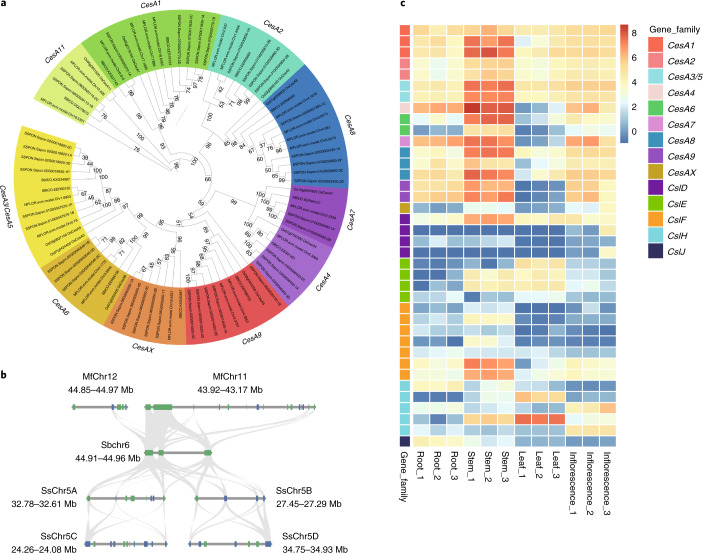
The overview of cellulose synthases in *M. floridulus*. **a**, Maximum likelihood phylogenetic tree of *CesA* genes from rice, sorghum, *M. floridulus* and *S. spontaneum*. Different colour ranges correspond to different *CesA* groups. Numbers at tree nodes represent bootstrap support values (1,000 replications). **b**, The syntenic relationship of *CslH* genes on Sbchr6, MfChr11, MfChr12 and Ss5ABCD. Grey lines indicate the homoeologous genes between any two syntenic regions. Note that there are 3, 5, 1, 3, 4, 2 and 2 homoeologues on the syntenic regions from Sbchr6, MfChr11, MfChr12, Ss5A, Ss5B, Ss5C and Ss5D, respectively. **c**, Heatmap of the expression level of *CesA/Csl* genes in different tissues (root, growing stem, mature leaf and inflorescence) of *M. floridulus*. The expression levels were shown as log_2_(TPM + 0.5). Only genes with average TPM > 1 across all tissues were shown as indicated in Supplementary Data 3.

*Miscanthus*, which has relatively high cellulose/hemicellulose content, is promising for use as a bioenergy feedstock^3,4,6,11^. To explore the molecular basis underlying this valuable trait, we identified the members of the *CesA/Csl* gene superfamily (except *CslA*/*CslC*; Methods) critical for cellulose/hemicellulose synthesis^16,18–20^ in the three lineages of Saccharinae. There are 35 *CesA/Csl* genes in *S. bicolor* (Supplementary Data 2), 77 in *M. floridulus* (Supplementary Data 3) and 109 in *S. spontaneum* (Supplementary Data 4). Phylogenetic analysis of the identified CesA/Csl proteins from the three Saccharinae species and rice revealed six groups, corresponding to six subfamilies of the rice CesA/Csl proteins (Supplementary Fig. 10). In light of these results, a phylogenetic tree of the CesA subfamily was then constructed and ten groups were identified with CesA3/CesA5 belonging to a same group and CesAX forming a new group not present in rice (Fig. 4a and Supplementary Note 3). On the basis of the synteny analysis and genomic coordinates, the *CesA/Csl* genes generally followed a 1:2 ratio in each single synteny block in *S. bicolor* versus two corresponding synteny blocks in *M. floridulus*, with a few exceptions. For example, there were five *CslH* genes on MfChr11 and only two *CslH* genes on MfChr12 syntenic to three tandem *CslH* genes on SbChr6 (Fig. 4b), suggestive of gene duplication and gene family expansion of the *CslH* subfamily in *M. floridulus*.

We then obtained the gene expression data of *CesA/Csl* for different tissues of mature *M. floridulus* plants from the RNA sequencing (RNA-seq) data (Supplementary Note 2). In general, the members of all *CesA* subfamilies except *CesA10* and *CesA11*, including *CesA1/3/5/6/8* which are expressed predominantly in primary cell walls and *CesA4/7/9* which are responsible mainly for cellulose synthesis in secondary cell walls, were expressed at the highest level in growing stems of all collected tissues (Fig. 4c). With respect to *Csl*s, one or several members of the *CslD*, *CslE*, *CslF* and *CslH* subfamilies were also highly expressed in growing stems and leaves (Fig. 4c), suggesting that active hemicellulose synthesis is occurring in these tissues. According to the histogram of expression level of all expressed genes (average transcripts per million mapped reads (TPM) > 1) in the stems (Supplementary Fig. 11), most expressed *CesAs* (11 of 17 genes) can be ranked among the top most expressed genes, suggestive of highly active cellulose synthesis; the same was true for several *Csl*s in the stems (one *CslD* and two *CslF*s) and leaves (one *CslH*) (Fig. 4c). Taken together, these observations hint that *Miscanthus* had a strong ability for cellulose/hemicellulose synthesis, which is in agreement with its promising use as a bioenergy feedstock.

### Population structure and diversity of the *Miscanthus* genus

To better understand the genetic diversity and population structure of the genus *Miscanthus*, we resequenced 74 diploid and tetraploid accessions distributed across China and one triploid *Miscanthus* × *giganteus*. These accessions include plants from *M. floridulus*, *M. sinensis*, *M. lutarioriparius* and *M. sacchariflorus*, as well as interspecific hybrids between them (Supplementary Table 12). After read mapping (Supplementary Data 5), variant calling and strict filtering (Supplementary Note 4), we identified 6.20 million high-confidence variants, which included 5,985,647 SNPs, 92,900 insertions and 122,070 deletions, with an average of 2.54 variants per kb (Fig. 1).

**Fig. 5:**
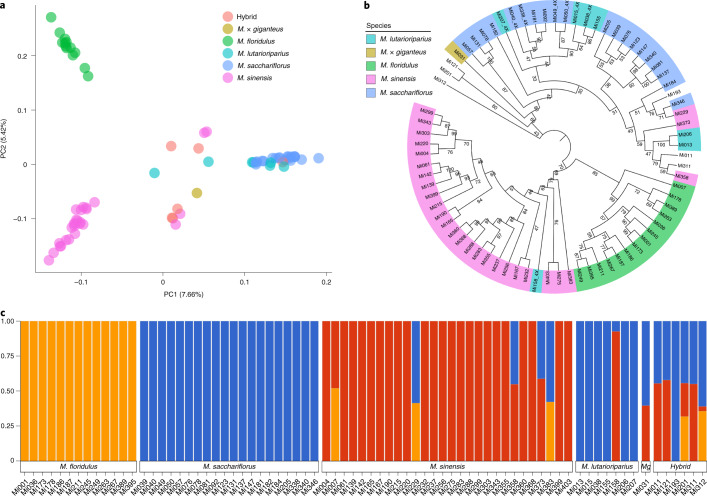
Population genetic structure and phylogenetic relationships among 75 *Miscanthus* accessions. **a**, Principal components (PCs) of accession variation. The percentage numbers in the brackets indicates the proportions of the two PCs. **b**, Bootstrapped tree of 75 *Miscanthus* accessions based on genetic distance. The tetraploid was labelled by the tail of the accession ID. **c**, ADMIXTURE plot for *Miscanthus*, showing the distribution of *K* = 3 genetic clusters with the smallest cross-validation error. Mg, *M.* × *giganteus*.

Principal component analysis (PCA) revealed three main clusters centred on *M. floridulus*, *M. sinensis* and *M. sacchariflorus*, encompassing most species, while the outliers consisted primarily of hybrids, *M.* × *giganteus* and some accessions of *M. sinensis* and *M. lutarioriparius* (Fig. 5a). The PCA results were supported by the phylogenetic tree constructed based on the SNP data (Fig. 5b), which showed ambiguity in the distinction between *M. lutarioriparius* and *M. sacchariflorus*, regardless of their differences in ploidy. Furthermore, *M.* × *giganteus* and other hybrids clustered very close to *M. sinensis* and *M. sacchariflorus*, which is consistent with the results of admixture-based analysis (Fig. 5c and Supplementary Fig. 12; for other *K*-values, see Supplementary Fig. 13). Notably, several accessions from *M. sinensis* and one accession from *M. lutarioriparius* did not cluster within their labelled species, although they were observed to be morphologically similar with their respective labelled species. Taken together, these observations suggest that most hybrids were probably derived from *M. sinensis* and *M. sacchariflorus*, whereas *M. lutarioriparius* is genetically similar to *M. sacchariflorus*. In addition, the fixation index (*F*_ST_) calculation of the different species showed that *M. sacchariflorus* and *M. lutarioriparius* were closely related, while *M. floridulus* was more distant from either of the two species (Supplementary Table 13). This increased phylogenetic distance from other accessions may be related to our observations of higher genetic diversity (higher *π* value) among the accessions of *M. floridulus* compared to other species (Supplementary Fig. 14), suggesting that the levels of diversity are generally low in the other three species, especially in *M. sacchariflorus* and *M. lutarioriparius*, although accessions of these two species were acquired from geographically distant locations (Supplementary Table 12).

## Discussion

After their divergence from the sorghum lineage, both *Miscanthus* and *Saccharum* underwent one or more rounds of independent WGD. Previous studies based on sequences from next-generation sequencing (NGS) of *Miscanthus* and expressed sequence tags (ESTs) sequences for *S. officinarum* suggested that an ancestor of these two genera also experienced a WGD before their evolutionary bifurcation. However, by identifying the paralogues in a recently released *S. spontaneum* reference genome and by plotting the distributions of their Ks values, we found that the Ks peak (0.012) of *S. spontaneum* was different from that of *S. officinarum* previously reported (0.04, on the basis of the So1-So2 paralogues). By using the high-quality reference genomes of *S. spontaneum*^15^ and *M. floridulus*, we found it implausible that *S. spontaneum* and *Miscanthus* shared a common tetraploid ancestor. Moreover, the difference in basic chromosome number between *S. officinarum* and *S. spontaneum* suggests that a very long time was needed between the divergence of these species and their ancestral divergence from *Miscanthus*; otherwise, there would not be high conservation of morphological and physiological traits of *S. officinarum* and *S. spontaneum*.

Subgenome dominance has often been reported in the genomes of allopolyploids especially in allopalaeopolyploids^14,37–39^. However, some recent allopolyploids, such as *Brassica napus*^41^, *Capsella bursa-pastoris*^42^ and *Triticum aestivum*^43^, display indistinguishable or slight subgenome dominance. In *M. floridulus*, only negligible subgenome dominance was detected, including insignificant differences in the ratio of gene loss between subgenomes and unbiased expression of syntelogues, both of which can make it difficult to draw a convincing inference of the nature of its polyploidy. On the basis of our present analyses, it is most likely that the ancestor *Miscanthus* underwent autopolyploidization. However, we can also suggest that the ancestor of *Miscanthus* may have experienced allopolyploidization at ~3.8–4.1 Ma or long after that time but the differentiation between the two subgenomes in *M. floridulus*, as observed in other allopolyploids^41–43^, appears to be undetectable due to the relatively short timeframe.

*CesA/Csl* genes play central roles in cellulose/hemicellulose synthesis, the speed and ability of which are positively correlated with their expression^19,44,45^. We measured their expression in different tissues of *Miscanthus* and found that, among all the expressed genes, most *CesA*s and a subset of *Csl*s showed extremely high expression in growing stems, which are a major source of biomass in *Miscanthus*^46^. Since WGD led to the duplication of most *CesA* genes and since each of the duplicated *CesA* genes was still highly expressed, the combined expression level of *CesAs* from the same subfamily and for the same function would be much higher than that of single *CesA*, resulting in increased synthesis of cellulose, as evidenced by the final content of cellulose in *Miscanthus*. Nevertheless, to reveal the relationship of the expression and number of *CesA/Csl* with the total cellulose/hemicellulose content, more well-designed experiments combining genetics, comparative genomics and transcriptomics are needed because of their importance in biofuel production.

By resequencing the 75 accessions from the genus *Miscanthus*, we provided a valuable genetic resource for *Miscanthus* breeding and improvement. Through the PCA, phylogenetic and structure analyses, this study also established that *M. sacchariflorus* and *M. lutarioriparius* are genomically indistinguishable from each other, validating the recent taxonomic evaluation and molecular results favouring the subspecies status of *M. lutarioriparius* belonging to *M. sacchariflorus*^47^. Moreover, there were some disagreements between morphology-based classification and phylogenetic and structure analysis-based classification of several accessions from *M. sinensis* and *M. lutarioriparius*, indicating that there was some hybridization or introgression among them, similar with the introgression among different subpopulations of another important bioenergy crop switchgrass^48^. These findings highlight the broad ability of different *Miscanthus* accessions to hybridize and further suggest that the boundaries between species may be less clear than previously thought. Additionally, crosses between *Miscanthus* and any member of the Saccharinae are also feasible and the ‘miscanes’ hybrids of *Saccharum* and *Miscanthus* have been used for sugarcane improvement^49^. With the completion of the reference genomes of three of the most economically important members of the Saccharinae, together with new tools for molecular breeding and transgenic research, we can envision the generation of a new hybrid that carries all of the desirable traits for bioenergy production from each species, such as high cellulose content and cold resistance from *Miscanthus*, drought tolerance from sorghum and disease resistance from sugarcane^3,4,6^.

## Methods

### Plant materials

The *M. floridulus* accession (‘MF70’, originally from Hunan Province in China) sequenced for genome assembly and annotations was grown in the field in Shandong Province, China. Accessions of different *Miscanthus* species and hybrids were also grown at the same location. Detailed information about them is listed in Supplementary Table 12.

### Genome sequencing

For PacBio single-molecule real-time (SMRT) sequencing, at least 10 μg of sheared DNA was used to construct 20-kb-insert-size libraries in accordance with the PacBio protocol. The libraries were then sequenced on a PacBio Sequel instrument. For the polishing of genome assembly and whole-genome resequencing using Illumina NGS, genomic DNA was extracted from young leaf tissue of the different accessions. Afterwards, 500-bp paired-end libraries were constructed using a NEBNext Ultra DNA Library Prep Kit and subsequently sequenced with the Illumina HiSeq 2500 platform.

### 10x Genomics library construction and sequencing

DNA sample was prepared, indexed and barcoded using a GemCode instrument (10x Genomics). Briefly, ~0.7 ng of 50-kb-long input DNA was used in GEM PCR together with 16-bp-long barcodes introduced in droplets. The droplets were then fractured and the intermediate DNA was purified and sheared to 500 bp for library construction. Libraries were subsequently sequenced via the Illumina HiSeq X Ten platform.

### Hi-C library construction and sequencing

The construction and sequencing of four Hi-C libraries was performed by the Annoroad Gene Technology Company. Briefly, young leaves of *M. floridulus* (accession ‘MF70’) were fixed with formaldehyde and then lysed, after which the cross-linked DNA was digested with MboI enzymes overnight. The enzymes were inactivated and cohesive ends were filled in by adding biotin-labelled dCTP. After proximity ligation in blunt-end ligation buffer, the cross-linking was reversed and DNA was purified for Hi-C library construction. The final library was sequenced on an Illumina HiSeq 2500 platform in 150-bp paired-end mode.

### Bionano optical genome maps construction

Bionano optical maps were generated using a Saphyr Genome Imaging Instrument in conjunction with DLE1 non-nicking enzyme (CTTAAG). Extracted high-molecular-weight DNA was nicked and labelled using the SaphyrPrep Kit, and the labelled DNA was then loaded for imaging into the Saphyr system (Bionano Genomics). Imaged molecules were subsequently assembled using Bionano Solve (v.3.3) and the Bionano alignment images of the assembly were visualized with Bionano Access (v.1.5.2, https://bionanogenomics.com/support/software-downloads/).

### Genome survey

The short reads used for polishing the genome assembly were quality filtered, after which the genome size, repeat sequence ratio and genome heterozygous rate were calculated using the *k*-mer method via Jellyfish^50^. The *k*-mer value used for the genome survey was 17.

### Genome assembly

Before assembly, PacBio reads that were longer than 7,000 bp were used as seed reads to correct read errors and generate consensus sequences; this was accomplished with the ‘daligner’ function in the main script of the FALCON assembler. After error correction produced highly accurate (up to 99.999%) consensus sequences, FALCON identified the overlaps between all the pairs of the pre-assembled error-corrected reads. The read overlaps were used to construct a directed string graph by following Myers’ algorithm. After the initial assembly, FALCON-Unzip was used to produce primary contigs (p-contigs) and haplotigs^31^. After phased polishing, FALCON-Phase^32^ then combined the primary contigs and haplotigs with the Hi-C data to extend phasing between unzipped haplotig blocks and to generate two phased assemblies (Supplementary Note 1). One of the phased assemblies was further polished with PacBio reads by the consensus-calling algorithm Quiver2 (ref. ^31^). The Illumina reads were also used to correct the contigs via Pilon3 (ref. ^51^). Heterozygosity was then removed from the error-corrected contigs using purge_haplotigs together with the Illumina clean reads. Afterwards, with the default settings, BWA software^52^ was used to align the 10x Genomics data to the assembly and fill-in gaps. Scaffolding was performed by fragScaff with the barcoded sequencing reads and the three-stages option as follows: stage1 “-m 3000 -q 30”; stage2 “-C 5”; stage3 “-j 1 -u 3” (ref. ^53^). These processes ultimately yielded the final draft of the genome assembly. For Hi-C-assisted genome assembly, the Hi-C clean data were aligned to the preceding assembly using HiC-Pro^54^ and Bowtie2 (ref. ^55^) software. Only read pairs with both reads in the pair-aligned contigs were considered valid gene pairs for scaffolding. By the use of linkage information and restriction enzyme sites, a string graph was used to construct the scaffold graph with LACHESIS4 (ref. ^56^).

### Linkage map construction

The mapping population comprised 116 F_1_ progeny generated from an interspecific cross between *M. floridulus* (MF70, 2*n* = 2*x* = 38) and *M. sacchariflorus* (MS62, 2*n* = 2*x* = 38). Genomic DNA was extracted from leaf tissue to construct restriction site-associated DNA sequencing (RAD-seq) libraries, which were then sequenced following standard protocols^57^. The generated reads were subsequently mapped to our genome assembly by BWA-MEM^52^ and variants were called by freebayes^58^ and then filtered by SnpSift^59^. A total of 6,472 SNPs were ultimately identified, including 5,827 parental testcross markers that were heterozygous only in *M. floridulus* (lm × ll) and 645 biparental markers that were heterozygous in both parents (hk × hk) according to cross-pollinator (CP) population type. A linkage map was constructed with JoinMap5 using the multipoint maximum likelihood mapping method^60^. A minimum independence logarithm of odds (LOD) score of 10 and a maximum recombination frequency of 0.4 were used to define linkage groups. In total, 3,799 SNPs clustered into 19 linkage groups covering 2,371.76 cM, with an average intermarker spacing of 0.65 cM. To validate the chromosome-scale assembly by Hi-C, the comparison of the genetic location and corresponding physical position of all SNP markers of the genetic map were assessed. In total, 92.74% of mapped markers were detected to be located at the same *M. floridulus* assembled chromosome. Of the markers, 5.35% were mapped equally well to homoeologous locations on the assembly (Supplementary Fig. 3).

### Repeat annotation

Tandem repeats were extracted using TRF (http://tandem.bu.edu/trf/trf.html)^61^ by ab initio prediction. For TE annotations, a combined strategy based on de novo structural identification and on a homology-based alignment search for whole-genome repeats was applied. A de novo repetitive elements database was built by combining the results of LTR_FINDER (http://tlife.fudan.edu.cn/tlife/ltr_finder/)^62^ and RepeatScout^63^ (http://www.repeatmasker.org/), with those of RepeatModeler (http://www.repeatmasker.org/RepeatModeler), set to default parameters. All repeat sequences with lengths >100 bp and <5% ‘N’ gaps constituted the raw TE library. Repeat regions were extracted with a homology-based repeat prediction using the Repbase (http://www.girinst.org/repbase) database using RepeatMasker (http://www.repeatmasker.org/) software and its in-house scripts (RepeatProteinMask) with default parameters. A custom non-redundant library that was generated by a combination of Repbase-based search and a de novo TE library, which was processed by uclust, was subjected to RepeatMasker for final repeat identification.

Specifically, to investigate LTR-RTs in detail, highly confident intact LTR-RTs were identified using the LTR_retriever^64^ pipeline, which integrated the results from LTR_FINDER^62^ and LTRharvest^65^ and efficiently removed false positives. The insertion time was then estimated as T = *K*/2*μ* (where *K* is the divergence distance between the 5′ LTR and 3′ LTR of intact LTR-RTs and where *μ* is 1.38 × 10^−8^ for grasses^66^).

### Gene model prediction and functional annotations

A combination of homology-based, ab initio and transcriptome-based gene prediction methods was used for protein-coding gene annotation. The protein sequences of six species (*Brachypodium distachyon, O. sativa, S. bicolor, Setaria italic, S. spontaneum* and *Z. mays*) were downloaded from the Ensembl plants database. The sequences were aligned to the genome using TBLASTN (v.2.2.26; *E* value ≤1 × 10^–5^), after which the matching proteins were aligned to homoeologous genome sequences for accurate spliced alignments via GeneWise (v.2.4.1)^67^, which was used to predict accurate gene structure within each protein region. For ab initio gene prediction, AUGUSTUS (v.3.2.3)^68^, GeneID (v.1.4)^69^, GeneScan (v.1.0)^70^, GlimmerHMM (v.3.04)^71^ and SNAP (https://github.com/KorfLab/SNAP) were used in our automated gene prediction pipeline. For genome annotation, RNA was extracted from five different tissues (root, leaf, stem, inflorescence and bud tissues) and mixed together equally for library construction. A de novo transcriptome assembly was then generated with Trinity (v.2.1.1)^72^, used for genome annotation and to optimize gene predictions. RNA-seq reads from different tissues were aligned to the genome using HISAT (v.2.0.4)^73^ with default parameters to identify exon regions and splice positions. The alignment results were then used as input by StringTie (v.1.3.3)^73^ with default parameters for genome-based transcript assembly. Finally, the non-redundant reference gene set was generated by merging genes predicted by the three methods above with EVidenceModeler (EVM; v.1.1.1)^74^ using PASA terminal exon support and including masked TEs as input for gene prediction.

Gene functions were assigned according to the best matches with Swiss-Prot protein sequences identified using BLASTP (with a threshold of *E* value ≤1 × 10^–5^). The motifs and domains were annotated using InterProScan (v.5.31)^75^ by searching against publicly available databases, including ProDom, PRINTS, Pfam, SMRT, PANTHER and PROSITE. The GO IDs for each gene were assigned according to the corresponding InterPro entry.

Further, transfer RNAs were predicted using the program tRNAscan-SE (http://lowelab.ucsc.edu/tRNAscan-SE/)^76^. Because ribosomal RNAs are highly conserved, we predicted rRNA sequences using BLAST with rRNAs from *S. bicolor* used as references. Other non-coding RNAs, including microRNAs and small nuclear RNAs, were identified by searching against the Rfam^77^ database via infernal software (http://infernal.janelia.org/)^78^ with the default parameters.

### Genome assembly assessment

Both BUSCO^34^ (http://busco.ezlab.org/) analysis and the CEGMA^33^ (http://korflab.ucdavis.edu/Datasets/cegma/) pipeline were used to assess the completeness of the genome assembly and annotations. Further, the LAI, which was implemented in the LTR_retriever package, was also applied to assess the genome assembly quality^35^.

### Gene family analysis

For gene family analysis, the online web server OrthoVenn2 (ref. ^79^) was used. Maize, sorghum, rice, *S. spontaneum* and *M. floridulus* protein sequences were used as inputs for online orthogroup clustering and annotations and GO enrichment.

### Genome synteny

Protein sequences from *M. floridulus*, *S. bicolor* and *S. spontaneum* were blasted against themselves using BLASTP (*E* value <1 × 10^–5^) to identify intergenome synteny blocks. The BLAST results were subjected to *c*-score filtering (*c*-score = 0.99) and the Python version JCVI (https://github.com/tanghaibao/jcvi) of MCScan was used to generate dot plots. MCScanX^80^ was used to determine synteny blocks with default parameters and at least five genes were required to define a synteny block. To identify intragenome synteny blocks, protein sequences from two homoeologous chromosomes were extracted to query against each other and after *c*-score filtering by JCVI, MCScanX was used to determine synteny blocks with default parameters. The Ks value was calculated via KaKs_Calculator^81^ for each gene pair in the aligned blocks. The distributions of all Ks values were plotted via the R software and ggplot2 package^82^. The peak Ks values were converted to divergence time^12^ according to the formula *T* = Ks/2*λ* (*T*, time; *λ*, average substitution rate) by using an average substitution rate of 6.5 × 10^−9^ for grasses^40^ to infer speciation or WGD events that occurred during the evolutionary history.

### Subgenome analysis

To analyse the gene loss of the two *M. floridulus* subgenomes, protein sequences of homoeologous chromosomes from *M. floridulus* and their corresponding homoeologous chromosome in *S. bicolor* were queried via BLAST against each other and synteny blocks were identified by MCScanX. Blocks shared by the three homoeologous chromosomes were then identified and syntelogues between any two of them were extracted for statistical analysis. For syntelogue expression analysis, RNA was extracted from leaf, growing stem, root and inflorescence tissues of mature *M. floridulus* plants and RNA-seq analysis was performed on these four tissues. There were three replicates for each tissue. The RNA-seq reads were quality filtered by fastp^83^ and then mapped to *M. floridulus* complementary DNA sequences by Salmon^84^. Meanwhile, TPM values were also calculated by Salmon. The expression of syntelogues across different tissues in each of homoeologous chromosome were retrieved and plotted via the R software.

### Analysis of *CesA/Csl* genes

All genes from the three species *S. bicolor*, *S. spontaneum* and *M. floridulus* were first annotated using InterProScan. The *CesA/Csl* genes were then retrieved from the files generated from the Interproscan-based functional annotation with Pfam accession ID ‘PF03552’. Because there were only five and nine *CslA/CslC* genes (described by two Pfams ‘PF00535’ and ‘PF13632’) identified in *S. bicolor* and *M. floridulus* and these genes distantly diverged from other *CesA/Csl* genes^16^, those genes were excluded from the downstream analysis. The gene coordinates were retrieved from the GFF files of the three genomes and the expression level of *CesA/Csl* genes in *M. floridulus* were obtained from the RNA-seq results of the previous subgenome analysis. The expression of *CesA/Csl* genes were retrieved and heatmap was drawn using the pheatmap package (https://CRAN.R-project.org/package=pheatmap) of R software with values of log_2_(TPM + 0.5).

### Resequencing and diversity analysis

DNA from different accessions of multiple *Miscanthus* species and hybrids was extracted for library construction and resequencing. The paired-end reads were quality filtered by fastp^83^ and subsequently mapped to the *M. floridulus* genome by BWA-MEM^52^. Via Picard, mapped reads were sorted and duplicated reads were marked. Uniquely mapped reads were then extracted from the sorted and duplicate-marked bam file with Sambamba^85^ using the parameter “-F ′′mapping_quality >= 30 and not (unmapped or secondary_alignment) and not ([XA] != null or [SA] != null)′′”. Then, SNPs and insertions/deletions (InDels) were called by freebayes^58^ using parameters “-standard-filters –min-coverage 4 –use-best-n-alleles 4 --genotype-qualities” and the polyploidy level of the different accessions were specified with “-A *Miscanthus*_freebayes_cnv_map.txt”. The variants were then filtered by a custom script to change low quality data (GQ < 30 or DP < 4 or DP > 50) to null and further filtered by SnpSift^59^ with the parameters “QUAL > 30 & NS >= 63 & (QUAL / AO > 10) & SAF > 0 & SAR > 0 & RPR > 1 & RPL > 1”. Afterwards, the multi-allelic genotype of polyploids were filtered and converted to bi-allelic genotype by our custom script and the remaining 6,200,617 variants were used in downstream analysis. PCA of the filtered variants was performed using the SMARTPCA program from EIGENSOFT (v.6.1.4)^86^. For phylogenetic analysis, SNPhylo^87^ software was used. Before tree construction, we filtered and pruned the SNPs with minor allele frequency (MAF) < 0.1, missing rate >0.1 and linkage disequilibrium (LD) threshold = 0.2. A total of 15,857 SNPs were ultimately selected for the SNPhylo pipeline. iTOL (v.5)^88^ was used to visualize the trees. For population structure analysis, the optimal ancestral population structure was estimated for the same variant set by using ADMIXTURE^89^ with ancestral population sizes *K* = 1–7 and choosing the population size with the smallest cross-validation error. On the basis of the *K* value, admixture analyses were then performed to infer population structure. Fixation index (*F*_ST_) and *π* value (indicating genetic diversity) were calculated by VCFtools^90^.

### Reporting Summary

Further information on research design is available in the Nature Research Reporting Summary linked to this article.

## Supplementary information

Supplementary Information Supplementary Figs. 1–13, Tables 1–14, Notes 1–4 and references.

Reporting Summary

Supplementary Data Supplementary Data 1–5.

## Data Availability

The genome assembly and gene annotation have been deposited in the NCBI database under BioProject number PRJNA598249 and BioSample number SAMN13702268. Source data are provided with this paper.

## Source data

Source Data Fig. 2 Statistical source data. Source Data Fig. 3 Statistical source data.

## Code availability

The custom codes used in this study are deposited in GitHub (https://github.com/sdysfhs319/Miscanthus_genome.git).

## References

[1] Paterson, A. H. *Genomics of the Saccharinae* (Springer, 2012).

[2] Przybysz K (2019). Production of sugar feedstocks for fermentation processes from selected fast growing grasses. Energies.

[3] van der Weijde T (2013). The potential of C4 grasses for cellulosic biofuel production. Front. Plant. Sci..

[4] de Siqueira Ferreira S, Nishiyama MY, Paterson AH, Souza GM (2013). Biofuel and energy crops: high-yield Saccharinae take center stage in the post-genomics era. Genome Biol..

[5] Waclawovsky AJ, Sato PM, Lembke CG, Moore PH, Souza GM (2010). Sugarcane for bioenergy production: an assessment of yield and regulation of sucrose content. Plant Biotechnol. J..

[6] Heaton EA, Dohleman FG, Long SP (2008). Meeting US biofuel goals with less land: the potential of *Miscanthus*. Glob. Change Biol..

[7] Arnoult S, Brancourt-Hulmel M (2014). A review on *Miscanthus* biomass production and composition for bioenergy use: genotypic and environmental variability and implications for breeding. Bioenergy Res..

[8] Rooney WL, Blumenthal J, Bean B, Mullet JE (2007). Designing sorghum as a dedicated bioenergy feedstock. Biofuels Bioprod. Biorefin..

[9] Pimentel LD, Batista VAP, Barros AFD, Teófilo RF, Dias LADS (2017). Chemical and bioenergetic characterization of sorghum agronomic groups. Pesqui. Agropecu. Trop..

[10] Hoang NV (2016). High-throughput profiling of the fiber and sugar composition of sugarcane biomass. Bioenergy Res..

[11] Lee W-C, Kuan W-C (2015). *Miscanthus* as cellulosic biomass for bioethanol production. Biotechnol. J..

[12] Kim C (2014). Comparative analysis of *Miscanthus* and *Saccharum* reveals a shared whole-genome duplication but different evolutionary fates. Plant Cell.

[13] Zhang K, Wang X, Cheng F (2019). Plant polyploidy: origin, evolution, and its influence on crop domestication. Hortic. Plant J..

[14] Cheng F (2018). Gene retention, fractionation and subgenome differences in polyploid plants. Nat. Plants.

[15] Zhang J (2018). Allele-defined genome of the autopolyploid sugarcane *Saccharum spontaneum* L. Nat. Genet..

[16] Little A (2018). Revised phylogeny of the cellulose synthase gene superfamily: insights into cell wall evolution. Plant Physiol..

[17] Yin, Y., Huang, J. & Xu, Y. The cellulose synthase superfamily in fully sequenced plants and algae. *BMC Plant Biol.* **9**, 99 (2009). 10.1186/1471-2229-9-99 PMC3091534 19646250

[18] Carpita NC (2011). Update on mechanisms of plant cell wall biosynthesis: how plants make cellulose and other (1→4)-β-d-glycans. Plant Physiol..

[19] Polko JK, Kieber JJ (2019). The regulation of cellulose biosynthesis in plants. Plant Cell.

[20] Sarkar P, Bosneaga E, Auer M (2009). Plant cell walls throughout evolution: towards a molecular understanding of their design principles. J. Exp. Bot..

[21] Tanaka K (2003). Three distinct rice cellulose synthase catalytic subunit genes required for cellulose synthesis in the secondary wall. Plant Physiol..

[22] Wang, L. et al. Expression profiling and integrative analysis of the CESA/CSL superfamily in rice. *BMC Plant Biol.* **10**, 282 (2010). 10.1186/1471-2229-10-282 PMC3022907 21167079

[23] Vega-Sanchez ME (2012). Loss of cellulose synthase-like F6 function affects mixed-linkage glucan deposition, cell wall mechanical properties, and defense responses in vegetative tissues of rice. Plant Physiol..

[24] Ermawar RA (2015). Distribution, structure and biosynthetic gene families of (1,3;1,4)-β-glucan in *Sorghum bicolor*. J. Integr. Plant Biol..

[25] Jobling SA (2015). Membrane pore architecture of the CslF6 protein controls (1-3,1-4)-β-glucan structure. Sci. Adv..

[26] Hu R (2017). Transcriptome analysis of genes involved in secondary cell wall biosynthesis in developing internodes of *Miscanthus lutarioriparius*. Sci. Rep..

[27] Zeng X (2020). Differential expression patterns reveal the roles of cellulose synthase genes (CesAs) in primary and secondary cell wall biosynthesis in *Miscanthus* × *giganteus*. Ind. Crops Prod..

[28] Deschamps S (2018). A chromosome-scale assembly of the sorghum genome using nanopore sequencing and optical mapping. Nat. Commun..

[29] Paterson AH (2009). The *Sorghum bicolor* genome and the diversification of grasses. Nature.

[30] Guo-te D (2013). Estimation of genome size of *Miscanthus floridulus*. J. Plant Genet. Res..

[31] Chin C-S (2016). Phased diploid genome assembly with single-molecule real-time sequencing. Nat. Methods.

[32] Kronenberg, Z. N. et al. Extended haplotype phasing of de novo genome assemblies with FALCON-Phase. Preprint at *bioRxiv*10.1101/327064 (2019).

[33] Parra G, Bradnam K, Korf I (2007). CEGMA: a pipeline to accurately annotate core genes in eukaryotic genomes. Bioinformatics.

[34] Simão FA, Waterhouse RM, Ioannidis P, Kriventseva EV, Zdobnov EM (2015). BUSCO: assessing genome assembly and annotation completeness with single-copy orthologs. Bioinformatics.

[35] Ou S, Chen J, Jiang N (2018). Assessing genome assembly quality using the LTR Assembly Index (LAI). Nucleic Acids Res..

[36] Swaminathan K (2012). A framework genetic map for *Miscanthus sinensis* from RNAseq-based markers shows recent tetraploidy. BMC Genom..

[37] Edger PP (2017). Subgenome dominance in an interspecific hybrid, synthetic allopolyploid, and a 140-year-old naturally established neo-allopolyploid monkeyflower. Plant Cell.

[38] Schnable JC, Springer NM, Freeling M (2011). Differentiation of the maize subgenomes by genome dominance and both ancient and ongoing gene loss. Proc. Natl Acad. Sci. USA.

[39] Zhao M, Zhang B, Lisch D, Ma J (2017). Patterns and consequences of subgenome differentiation provide insights into the nature of paleopolyploidy in plants. Plant Cell.

[40] Gaut BS, Morton BR, McCaig BC, Clegg MT (1996). Substitution rate comparisons between grasses and palms: synonymous rate differences at the nuclear gene *Adh* parallel rate differences at the plastid gene *rbcL*. Proc. Natl Acad. Sci. USA.

[41] Chalhoub B (2014). Early allopolyploid evolution in the post-Neolithic *Brassica napus* oilseed genome. Science.

[42] Douglas GM (2015). Hybrid origins and the earliest stages of diploidization in the highly successful recent polyploid *Capsella bursa-pastoris*. Proc. Natl Acad. Sci. USA.

[43] Consortium IWGS (2014). A chromosome-based draft sequence of the hexaploid bread wheat (*Triticum aestivum*) genome. Science.

[44] Xie L, Yang C, Wang X (2011). Brassinosteroids can regulate cellulose biosynthesis by controlling the expression of CESA genes in *Arabidopsis*. J. Exp. Bot..

[45] Kim WC (2013). MYB46 directly regulates the gene expression of secondary wall-associated cellulose synthases in *Arabidopsis*. Plant J..

[46] Bergs M (2020). Comparing chemical composition and lignin structure of *Miscanthus* × *giganteus* and *Miscanthus nagara* harvested in autumn and spring and separated into stems and leaves. RSC Adv..

[47] Clark LV (2019). Population structure of *Miscanthus sacchariflorus* reveals two major polyploidization events, tetraploid-mediated unidirectional introgression from diploid *M. sinensis*, and diversity centred around the Yellow Sea. Ann. Bot..

[48] Lovell JT (2021). Genomic mechanisms of climate adaptation in polyploid bioenergy switchgrass. Nature.

[49] Chen Y, Lo C (1989). Disease resistance and sugar content in *Saccharum–Miscanthus* hybrids. Taiwan Sugar.

[50] Marçais G, Kingsford C (2011). A fast, lock-free approach for efficient parallel counting of occurrences of *k*-mers. Bioinformatics.

[51] Walker BJ (2014). Pilon: an integrated tool for comprehensive microbial variant detection and genome assembly improvement. PLoS ONE.

[52] Li, H. Aligning sequence reads, clone sequences and assembly contigs with BWA-MEM. Preprint at https://arxiv.org/pdf/1303.3997.pdf (2013).

[53] Mostovoy Y (2016). A hybrid approach for *de novo* human genome sequence assembly and phasing. Nat. Methods.

[54] Servant N (2015). HiC-Pro: an optimized and flexible pipeline for Hi-C data processing. Genome Bio..

[55] Langmead B, Salzberg SL (2012). Fast gapped-read alignment with Bowtie 2. Nat. Methods.

[56] Burton JN (2013). Chromosome-scale scaffolding of *de novo* genome assemblies based on chromatin interactions. Nat. Biotechnol..

[57] DaCosta JM, Sorenson MD (2014). Amplification biases and consistent recovery of loci in a double-digest RAD-seq protocol. PLoS ONE.

[58] Garrison, E. & Marth, G. Haplotype-based variant detection from short-read sequencing. Preprint at https://export.arxiv.org/abs/1207.3907v2 (2012).

[59] Ruden DM (2012). Using *Drosophila melanogaster* as a model for genotoxic chemical mutational studies with a new program, SnpSift. Front. Genet..

[60] Van Ooijen J (2011). Multipoint maximum likelihood mapping in a full-sib family of an outbreeding species. Genet. Res..

[61] Benson G (1999). Tandem repeats finder: a program to analyze DNA sequences. Nucleic Acids Res..

[62] Xu Z, Wang H (2007). LTR_FINDER: an efficient tool for the prediction of full-length LTR retrotransposons. Nucleic Acids Res..

[63] Price AL, Jones NC, Pevzner PA (2005). De novo identification of repeat families in large genomes. Bioinformatics.

[64] Ou S, Jiang N (2018). LTR_retriever: a highly accurate and sensitive program for identification of long terminal repeat retrotransposons. Plant Physiol..

[65] Ellinghaus D, Kurtz S, Willhoeft U (2008). LTRharvest, an efficient and flexible software for de novo detection of LTR retrotransposons. BMC Bioinform..

[66] Ma J, Devos KM, Bennetzen JL (2004). Analyses of LTR-retrotransposon structures reveal recent and rapid genomic DNA loss in rice. Genome Res..

[67] Birney E, Clamp M, Durbin R (2004). GeneWise and genomewise. Genome Res..

[68] Stanke M (2006). AUGUSTUS: ab initio prediction of alternative transcripts. Nucleic Acids Res..

[69] Blanco E, Abril JF (2009). Computational gene annotation in new genome assemblies using GeneID. Methods Mol. Biol..

[70] Lynn AM (2001). An automated annotation tool for genomic DNA sequences using GeneScan and BLAST. J. Genet..

[71] Majoros WH, Pertea M, Salzberg SL (2004). TigrScan and GlimmerHMM: two open source ab initio eukaryotic gene-finders. Bioinformatics.

[72] Grabherr MG (2011). Trinity: reconstructing a full-length transcriptome without a genome from RNA-Seq data. Nat. Biotechnol..

[73] Pertea M, Kim D, Pertea GM, Leek JT, Salzberg SL (2016). Transcript-level expression analysis of RNA-seq experiments with HISAT, StringTie and Ballgown. Nat. Protoc..

[74] Haas BJ (2008). Automated eukaryotic gene structure annotation using EVidenceModeler and the Program to Assemble Spliced Alignments. Genome Biol..

[75] Zdobnov EM, Apweiler R (2001). InterProScan—an integration platform for the signature-recognition methods in InterPro. Bioinformatics.

[76] Lowe TM, Eddy SR (1997). tRNAscan-SE: a program for improved detection of transfer RNA genes in genomic sequence. Nucleic Acids Res..

[77] Griffiths-Jones S (2005). Rfam: annotating non-coding RNAs in complete genomes. Nucleic Acids Res..

[78] Nawrocki EP, Eddy SR (2013). Infernal 1.1: 100-fold faster RNA homology searches. Bioinformatics.

[79] Xu L (2019). OrthoVenn2: a web server for whole-genome comparison and annotation of orthologous clusters across multiple species. Nucleic Acids Res..

[80] Wang, Y. et al. MCScanX: a toolkit for detection and evolutionary analysis of gene synteny and collinearity. *Nucleic Acids Res.* **40**, e49 (2012). 10.1093/nar/gkr1293 PMC3326336 22217600

[81] Zhang Z (2006). KaKs_Calculator: calculating Ka and Ks through model selection and model averaging. Genomics Proteomics Bioinformatics.

[82] Wickham, H. *ggplot2: Elegant Graphics for Data Analysis* (Springer-Verlag, 2016).

[83] Chen S, Zhou Y, Chen Y, Gu J (2018). fastp: an ultra-fast all-in-one FASTQ preprocessor. Bioinformatics.

[84] Patro R, Duggal G, Love MI, Irizarry RA, Kingsford C (2017). Salmon provides fast and bias-aware quantification of transcript expression. Nat. Methods.

[85] Tarasov A, Vilella AJ, Cuppen E, Nijman IJ, Prins P (2015). Sambamba: fast processing of NGS alignment formats. Bioinformatics.

[86] Patterson N, Price AL, Reich D (2006). Population structure and eigenanalysis. PLoS Genet..

[87] Lee T-H, Guo H, Wang X, Kim C, Paterson AH (2014). SNPhylo: a pipeline to construct a phylogenetic tree from huge SNP data. BMC Genomics.

[88] Letunic I, Bork P (2016). Interactive tree of life (iTOL) v3: an online tool for the display and annotation of phylogenetic and other trees. Nucleic Acids Res..

[89] Alexander DH, Novembre J, Lange K (2009). Fast model-based estimation of ancestry in unrelated individuals. Genome Res..

[90] Danecek P (2011). The variant call format and VCFtools. Bioinformatics.

