# Author Correction: The reference genome of *Miscanthus floridulus* illuminates the evolution of Saccharinae

**DOI:** 10.1038/s41477-021-00972-4

**Published:** 2021-06-28

**Authors:** Guobin Zhang, Chunxia Ge, Pingping Xu, Shukai Wang, Senan Cheng, Yanbin Han, Yancui Wang, Yongbin Zhuang, Xinwei Hou, Ting Yu, Xitong Xu, Shuhan Deng, Quanquan Li, Yinqing Yang, Xiaoru Yin, Weidong Wang, Wenxue Liu, Chunxiao Zheng, Xuezhen Sun, Zhenlin Wang, Ray Ming, Shuting Dong, Jianxin Ma, Xiansheng Zhang, Cuixia Chen

**Affiliations:** 1 State Key Laboratory of Crop Biology, Shandong Agricultural University, Taian, China; 2 College of Agronomy, Shandong Agricultural University, Taian, China; 3 Novogene Bioinformatics Institute, Beijing, China; 4 Department of Plant Biology, University of Illinois at Urbana-Champaign, Urbana, IL USA; 5 Department of Agronomy, Purdue University, West Lafayette, IN USA

**Keywords:** Genomics, Biofuels, Plant evolution

Correction to: *Nature Plants* 10.1038/s41477-021-00908-y, published online 6 May 2021.

In Fig. 5c of this Article originally published, the *Miscanthus* accession names listed along the *x* axis were outdated. They have now been updated to match the accession names shown in Supplementary Table 12 and Fig. 5b, and the corrected panel is shown below.

**Fig. 5c:**
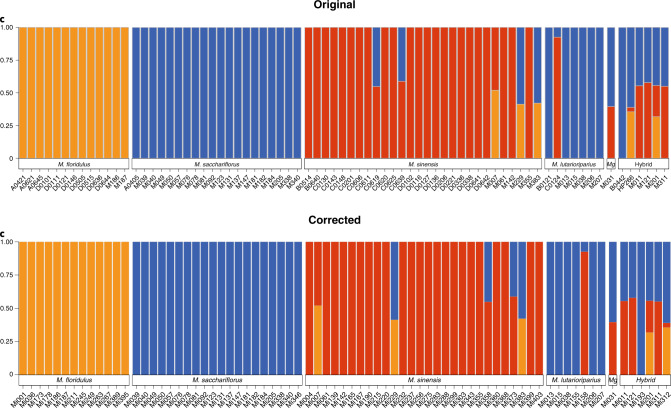
Original and Corrected.

