# Publisher Correction: The reference genome of *Miscanthus floridulus* illuminates the evolution of Saccharinae

**DOI:** 10.1038/s41477-021-00968-0

**Published:** 2021-06-28

**Authors:** Guobin Zhang, Chunxia Ge, Pingping Xu, Shukai Wang, Senan Cheng, Yanbin Han, Yancui Wang, Yongbin Zhuang, Xinwei Hou, Ting Yu, Xitong Xu, Shuhan Deng, Quanquan Li, Yinqing Yang, Xiaoru Yin, Weidong Wang, Wenxue Liu, Chunxiao Zheng, Xuezhen Sun, Zhenlin Wang, Ray Ming, Shuting Dong, Jianxin Ma, Xiansheng Zhang, Cuixia Chen

**Affiliations:** 1 State Key Laboratory of Crop Biology, Shandong Agricultural University, Taian, China; 2 College of Agronomy, Shandong Agricultural University, Taian, China; 3 Novogene Bioinformatics Institute, Beijing, China; 4 Department of Plant Biology, University of Illinois at Urbana-Champaign, Urbana, IL USA; 5 Department of Agronomy, Purdue University, West Lafayette, IN USA

**Keywords:** Genomics, Biofuels, Plant evolution

Correction to: *Nature Plants* 10.1038/s41477-021-00908-y, published online 6 May 2021.

This Article was originally published with an incorrect copyright status, and should have been Open Access. This error has now been corrected and the Article is now published under a Creative Commons licence CC BY 4.0.

