# Supplementary material for: The reference genome of Miscanthus floridulus illuminates the evolution of Saccharinae
Source: Nat Plants. 2021 May 6;7(5):608–18. doi: 10.1038/s41477-021-00908-y (PMC8238680; doi:10.1038/s41477-021-00908-y)
Supplement: Supplementary file 1 — Supplementary Figs. 1–13, Tables 1–14, Notes 1–4 and references. [file 41477_2021_908_MOESM1_ESM.pdf]

nature portfolio

https://doi.org/10.1038/s41477-021-00908-y

## Supplementary information

# The reference genome of *Miscanthus floridulus* illuminates the evolution of Saccharinae

In the format provided by the authors and unedited

## Supplementary Table 1. Genome survey results of *M. floridulus*

| Kmer | Depth | n_kmer | Genome_size(Mbp) | Revised Genome_size(M) | Heterozygous rate (%) | Repeat rate (%) |
|---|---|---|---|---|---|---|
| 17 | 88 | 218,501,920,900 | 2482.98 | 2462.35 | 1.89 | 73.42 |

**Supplementary Table 2. The summary of genome assembly results of *M. floridulus.***

| **Genomic features** | ***M. floridulus*** |
|---|---|
| Estimated genome size (Mb) | 2,462.35 |
| Length of genome assembly (Mb) | 2,684.39 |
| Largest scaffolds (bp) | 11,118,994 |
| Scaffold N50 (bp) | 1,644,988 |
| Largest contig (bp) | 6,928,031 |
| Contig N50 (bp) | 820,435 |
| Sequences anchored to chromosomes (%) | 91.03 |
| Numbers of gene models | 76,913 |
| Mean gene length (bp) | 3,261.4 |
| Mean coding sequence length (bp) | 1,014.4 |
| Total size of transposable elements (bp) | 1,890,088,665 |

**Supplementary Table 3. Summary of Bionano physical maps aligned to *M. floridulus* genomes**

| | ***M. floridulus*** |
|---|---|
| **Bionano molecule stats** | |
| Enzyme | DLE1 |
| Total length (Gb,>=150 kbp) | 1,188.73 |
| Molecule N50 (Kb,>=150 kbp) | 277.50 |
| Label density (/100kb,>=150 kbp) | 13.67 |
| **Bionano assembly results** | |
| Total genome map number | 323.00 |
| Total genome map length (Mbp) | 4,618.41 |
| Map contig N50 (Mbp) | 49.31 |
| Aligned length (Mbp) | 2,778.15 |
| Unique aligned length* (Mbp) | 2,453.40 |
| Unique aligned / reference Length (%) | 91.40 |

* The "Unique aligened length" value produced by ex_informaticsReport in Bionano Solve Software

**Supplementary Table 4. Completeness of the genome based on CEGMA**

| Description | Fully mapped CEGs (%completeness) | Fully and partially mapped CEGs (%completeness) |
|---|---|---|
| Number (percentage) | 224 (90.32%) | 238 (95.97%) |

**Supplementary Table 5. Completeness of the genome based on BUSCO analysis**

| Description | Number | Percentage |
|---|---|---|
| Complete BUSCOs (C) | 1378 | 95.7% |
| single-copy BUSCOs (S) | 495 | 34.4% |
| duplicated BUSCOs (D) | 883 | 61.3% |
| Fragmented BUSCOs (F) | 11 | 0.8% |
| Missing BUSCOs (M) | 50 | 3.5% |
| Total BUSCO groups searched | 1440 | 100% |

**Supplementary Table 6. Assessment of genome sequence consistency**

| | | Percentage |
|---|---|---|
| Reads | Mapping rate (%) | 98.41 |
| | Unique mapping rate (%) | 64.32 |
| Genome | Average sequencing depth | 88.47 |
| | Coverage (%) | 99.81 |
| | Unique mapping coverage (%) | 93.16 |
| | Coverage at least 4X (%) | 99.69 |
| | Coverage at least 10X (%) | 99.50 |
| | Coverage at least 20X (%) | 99.15 |

**Supplementary Table 7. Statistics of repeat sequences in *M. floridulus* genome**

| | Number | Total size (bp) | Percentage of genome assembly (%) |
|---|---|---|---|
| **Class I: Retrotransposon** | 1,546,529 | 1,529,418,766 | 56.97 |
| **LTR Retrotransposon** | 1,497,779 | 1,497,316,138 | 55.78 |
| Copia | 422,848 | 382,074,553 | 14.23 |
| Gypsy | 621,771 | 840,098,311 | 31.30 |
| Unknown | 453,160 | 275,143,274 | 10.25 |
| **Non-LTR Retrotransposon** | 48,750 | 32,102,628 | 1.20 |
| SINE | 1,879 | 554,790 | 0.02 |
| LINE | 46,871 | 31,547,838 | 1.18 |
| **Class II: DNA transposon** | 51,447 | 177,282,866 | 6.60 |
| **Helitron** | 7,825 | 59,022,774 | 2.20 |
| **TIR** | 43,622 | 118,260,092 | 4.41 |
| DTA hAT | 3,354 | 2,149,350 | 0.08 |
| DTC CACTA | 3,927 | 12,872,513 | 0.48 |
| DTH Pif/Harbinger | 3,641 | 4,769,420 | 0.18 |
| DTM Mutator | 11,215 | 31,920,680 | 1.19 |
| DTT Tc1/Mariner | 21,485 | 66,548,129 | 2.48 |
| **Tandem Repeats** | 810,680 | 183,387,033 | 6.83 |
| **Total** | 2,408,656 | 1,890,088,665 | 70.41 |

**Supplementary Table 8. Go enrichment of gene models unique to *M. floridulus***

| GO ID | Namespace | Name | Count | p-value* |
|---|---|---|---|---|
| GO:0032196 | biological_process | transposition | 17 | 1.61E-19 |
| GO:0009791 | biological_process | post-embryonic development | 17 | 7.23E-19 |
| GO:0003964 | molecular_function | RNA-directed DNA polymerase activity | 9 | 9.44E-10 |
| GO:0004721 | molecular_function | phosphoprotein phosphatase activity | 10 | 5.32E-06 |
| GO:2000280 | biological_process | regulation of root development | 5 | 6.48E-05 |
| GO:0006260 | biological_process | DNA replication | 4 | 0.00041001 |
| GO:0002229 | biological_process | defense response to oomycetes | 4 | 0.001123419 |
| GO:0004523 | molecular_function | RNA-DNA hybrid ribonuclease activity | 3 | 6.36E-05 |

*: To compute the p values, hypergeometric test was used.

**Supplementary Table 9. Go enrichment of gene models shared by sorghum, *M. floridulus* and *S. spontaneum***

| GO ID | Namespace | Name | Count | p-value* |
|---|---|---|---|---|
| GO:0010411 | biological_process | xyloglucan metabolic process | 13 | 3.48E-05 |
| GO:0016042 | biological_process | lipid catabolic process | 31 | 3.56E-05 |
| GO:0004672 | molecular_function | protein kinase activity | 10 | 9.68E-05 |
| GO:0006355 | biological_process | regulation of transcription, DNA-templated | 26 | 0.000565 |
| GO:0006970 | biological_process | response to osmotic stress | 11 | 1.03E-05 |

*: To compute the p values, hypergeometric test was used.

**Supplementary Table 10. The corresponding region of homologous chromosomes based on syntenic relationship**

| MfChr8 | MfChr7 | Sbchr4 | Sbchr7 | MfChf13 |
|---|---|---|---|---|
| 0-45.9 Mb | 0-57.8 Mb | 0-20.8 Mb | | |
| 74.3-44.4 Mb | | | 0-23.8 Mb | 0-43.5 Mb |
| 74.9-134.6 Mb | | | 36.4-65.5 Mb | 43.7-90.0 Mb |
| 134.3-226.8 Mb | 59.4-133.5 Mb | 38.5-68.5 Mb | | |

**Supplementary Table 11. The gene loss of syntelogs between two subgenomes of *M. floridulus***

| | syntelogs between MfChrA and Sb | syntelogs between MfChrB and Sb | Lost syntelogs in MfChrA compared with both Sb and MfChrB | The percentage of lost syntelogs in MfChrA compared with both Sb and MfChrB | Lost syntelogs in MfChrB compared with both Sb and MfChrA | The percentage of lost syntelogs in MfChrB compared with both Sb and MfChrA |
|---|---|---|---|---|---|---|
| MfChr1-Sb1-MfChr2 | 2,903 | 2,721 | 944 | 24.7% | 1,086 | 28.4% |
| MfChr3-Sb2-MfChr4 | 2,203 | 2,028 | 595 | 21.3% | 770 | 27.5% |
| MfChr5-Sb3-MfChr6 | 2,365 | 2,301 | 798 | 25.4% | 822 | 26.2% |
| MfChr7-Sb4-MfChr8 | 1,861 | 2,021 | 705 | 27.1% | 545 | 20.9% |
| MfChr9-Sb5-MfChr10 | 691 | 760 | 354 | 33.0% | 285 | 26.6% |
| MfChr11-Sb6-MfChr12 | 1,528 | 1,461 | 331 | 16.9% | 358 | 18.3% |
| MfChr13-Sb7-MfChr8 | 1,053 | 1,115 | 338 | 23.5% | 276 | 19.2% |
| MfChr14-Sb8-MfChr15 | 746 | 1,458 | 1,055 | 58.3% | 343 | 18.9% |
| MfChr16-Sb9-MfChr17 | 1,285 | 1,282 | 469 | 26.6% | 467 | 26.5% |
| MfChr18-Sb10-MfChr19 | 1,358 | 1,334 | 447 | 24.4% | 471 | 25.7% |
| Total (except for MfChr14-Sb8-MfChr15) | 15,247 | 15,023 | 4,981 | 24.4% | 5,080 | 24.9% |

**Supplementary Table 12. The detailed information of accessions for resequencing**

| ID | Species | Original location | Ploidy level | Latitude (°) | Longitude (°) |
|---|---|---|---|---|---|
| Mi001 | *M. floridulus* | Hunan | diploid | N25.78 | E113.02 |
| Mi004 | *M. sinensis* | Hunan | diploid | N29.58 | E111.38 |
| Mi007 | *M. sinensis* | Hunan | diploid | N28.25 | E113.08 |
| Mi011 | Hybrid | Hunan | diploid | N28.28 | E112.55 |
| Mi013 | *M. lutarioriparius* | Hunan | diploid | N28.28 | E112.55 |
| Mi015 | *M. lutarioriparius* | Hunan | tetraploid | N28.28 | E112.55 |
| Mi031 | *M.×giganteus* | Illinois,America | triploid | N40.06 | W88.13 |
| Mi036 | *M. floridulus* | Jiangsu | diploid | N32.52 | E120.15 |
| Mi038 | *M. lutarioriparius* | Jiangsu | tetraploid | N32.06 | E118.76 |
| Mi039 | *M. sacchariflorus* | Jiangsu | diploid | N32.05 | E118.92 |
| Mi040 | *M. sacchariflorus* | Shandong | tetraploid | N36.20 | E117.11 |
| Mi049 | *M. sacchariflorus* | Henan | tetraploid | N34.63 | E112.41 |
| Mi050 | *M. sacchariflorus* | Henan | tetraploid | N34.82 | E112.31 |
| Mi057 | *M. sacchariflorus* | Hebei | diploid | N38.31 | E113.98 |
| Mi061 | *M. sinensis* | Shandong | diploid | N36.22 | E117.10 |
| Mi076 | *M. sacchariflorus* | Shandong | diploid | N37.83 | E119.00 |
| Mi078 | *M. sacchariflorus* | Shanxi | diploid | N34.83 | E110.39 |
| Mi081 | *M. sacchariflorus* | Shaanxi | diploid | N34.36 | E107.88 |
| Mi092 | *M. sacchariflorus* | Ningxia | diploid | N38.31 | E106.21 |
| Mi121 | Hybrid | Guizhou | diploid | N27.52 | E106.49 |
| Mi123 | *M. sacchariflorus* | Heilongjiang | diploid | N46.02 | E128.55 |
| Mi131 | *M. sacchariflorus* | Heilongjiang | diploid | N47.12 | E123.38 |
| Mi137 | *M. sacchariflorus* | Liaoning | diploid | N41.70 | E123.36 |
| Mi139 | *M. sinensis* | Liaoning | diploid | N23.56 | E122.20 |
| Mi142 | *M. sinensis* | Jilin | diploid | N43.77 | E127.32 |
| Mi147 | *M. sacchariflorus* | Jilin | diploid | N43.77 | E127.32 |
| Mi155 | *M. lutarioriparius* | Jiangxi | diploid | N29.73 | E116.22 |
| Mi158 | *M. lutarioriparius* | Jiangxi | tetraploid | N28.37 | E116.26 |
| Mi165 | *M. sinensis* | Jiangxi | diploid | N28.27 | E117.56 |
| Mi167 | *M. sinensis* | Jiangxi | diploid | N27.55 | E116.44 |
| Mi173 | *M. floridulus* | Jiangxi | diploid | N25.53 | E116.01 |
| Mi178 | *M. floridulus* | Jiangxi | diploid | N28.27 | E117.56 |
| Mi181 | *M. sacchariflorus* | Shandong | diploid | N37.48 | E121.45 |
| Mi182 | *M. sacchariflorus* | Shandong | tetraploid | N37.51 | E121.44 |
| Mi184 | *M. sacchariflorus* | Anhui | diploid | N31.92 | E117.21 |
| Mi186 | *M. floridulus* | Anhui | diploid | N32.38 | E116.79 |
| Mi187 | *M. floridulus* | Anhui | diploid | N31.75 | E117.55 |
| Mi190 | *M. sinensis* | Anhui | diploid | N30.41 | E118.24 |
| Mi193 | Hybrid | Anhui | diploid | N30.70 | E118.40 |

| | | | | | |
|---|---|---|---|---|---|
| Mi201 | Hybrid | Hubei | diploid | N30.52 | E114.35 |
| Mi205 | *M. sacchariflorus* | Hubei | diploid | N30.55 | E114.40 |
| Mi206 | *M. lutarioriparius* | Hubei | diploid | N30.53 | E114.41 |
| Mi207 | *M. lutarioriparius* | Hubei | tetraploid | N30.52 | E114.40 |
| Mi211 | *M. floridulus* | Hubei | diploid | N29.72 | E113.88 |
| Mi215 | *M. sinensis* | Hubei | diploid | N31.15 | E109.56 |
| Mi220 | *M. sinensis* | Hubei | diploid | N30.70 | E111.30 |
| Mi229 | *M. sinensis* | Guangdong | diploid | N23.60 | E113.22 |
| Mi232 | *M. sinensis* | Guangdong | diploid | N24.46 | E113.16 |
| Mi237 | *M. sinensis* | Guangdong | diploid | N21.51 | E111.58 |
| Mi245 | *M. floridulus* | Guangdong | diploid | N22.32 | E114.03 |
| Mi249 | *M. floridulus* | Guangdong | diploid | N22.47 | E115.22 |
| Mi256 | *M. sinensis* | Hainan | diploid | N19.36 | E110.10 |
| Mi263 | *M. floridulus* | Fujian | diploid | N25.43 | E119.22 |
| Mi267 | *M. floridulus* | Fujian | diploid | N26.04 | E119.17 |
| Mi275 | *M. sinensis* | Fujian | diploid | N26.04 | E119.17 |
| Mi283 | *M. sinensis* | Guangxi | diploid | N23.23 | E110.04 |
| Mi288 | *M. sinensis* | Guangxi | diploid | N22.49 | E108.21 |
| Mi299 | *M. sinensis* | Yunnan | diploid | N21.48 | E101.56 |
| Mi303 | *M. sinensis* | Yunnan | diploid | N21.95 | E100.50 |
| Mi311 | Hybrid | Hunan | diploid | N27.51 | E111.53 |
| Mi312 | Hybrid | Hunan | diploid | N27.51 | E111.53 |
| Mi338 | *M. sacchariflorus* | Shandong | tetraploid | N37.30 | E122.06 |
| Mi340 | *M. sacchariflorus* | Shaanxi | diploid | N33.54 | E106.31 |
| Mi343 | *M. sinensis* | Shaanxi | diploid | N33.93 | E106.51 |
| Mi346 | *M. sacchariflorus* | Gansu | diploid | N35.33 | E107.37 |
| Mi355 | *M. sinensis* | Guizhou | diploid | N26.22 | E108.05 |
| Mi358 | *M. sinensis* | Guizhou | diploid | N26.33 | E106.28 |
| Mi360 | *M. sinensis* | Guizhou | diploid | N26.24 | E106.40 |
| Mi368 | *M. sinensis* | Sichuan | diploid | N29.33 | E103.45 |
| Mi373 | *M. sinensis* | Sichuan | diploid | N29.20 | E104.46 |
| Mi383 | *M. sinensis* | Shandong | diploid | N36.04 | E117.13 |
| Mi389 | *M. floridulus* | Zhejiang | diploid | N27.50 | E121.09 |
| Mi395 | *M. floridulus* | Zhejiang | diploid | N28.07 | E119.34 |
| Mi399 | *M. sinensis* | Zhejiang | diploid | N29.59 | E122.12 |
| Mi403 | *M. sinensis* | Zhejiang | diploid | N28.35 | E119.16 |

**Supplementary Table 13. The $F_{ST}$ value between any two species**

| | *M. lutarioriparius* | *M. floridulus* | *M. sinensis* |
|---|---|---|---|
| *M. floridulus* | 0.083038 | | |
| *M. sinensis* | 0.031393 | 0.032476 | |
| *M. sacchariflorus* | 0.022728 | 0.12069 | 0.050142 |

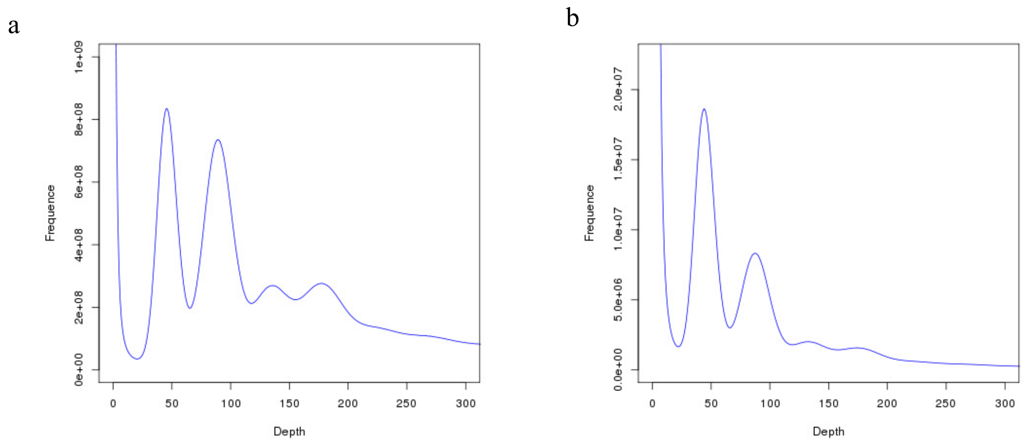


**Supplementary Figure 1. k-mer based genome survey**

(A) The frequency plot of k-mer number and depth given that k-mer (k=17). (B) The frequency plot of k-mer types and depth given that k=17.

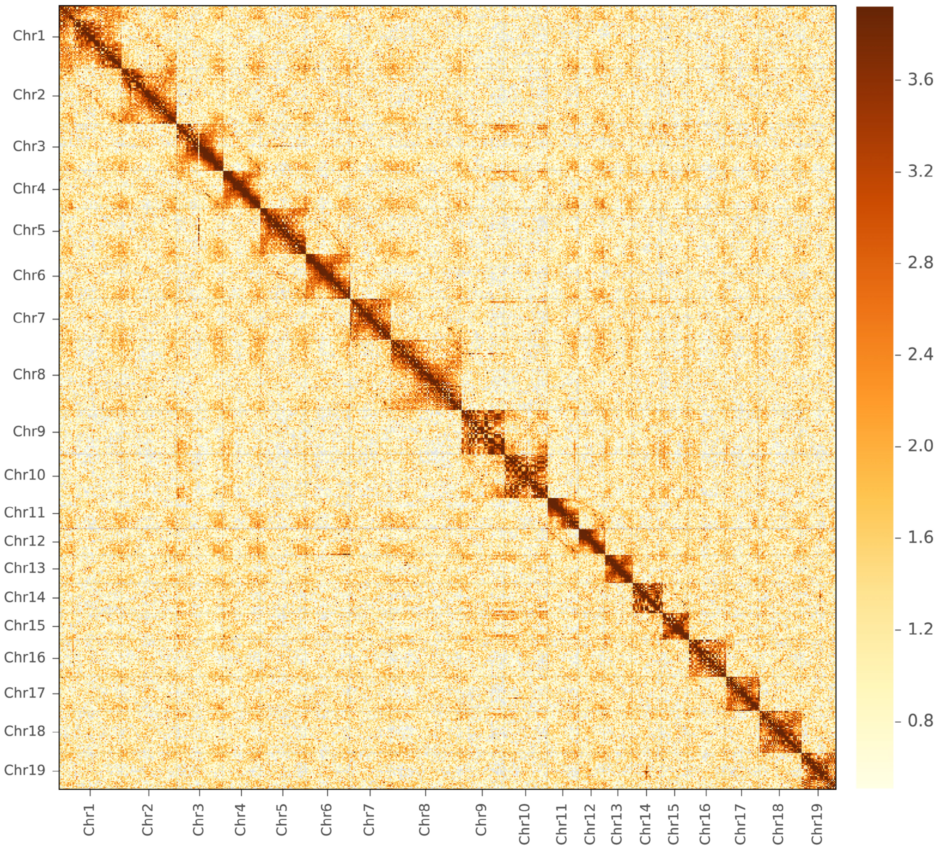


**Supplementary Figure 2. Genome-wide analysis of chromatin interactions at 500-kb resolution in *M. floridulus* genome.**

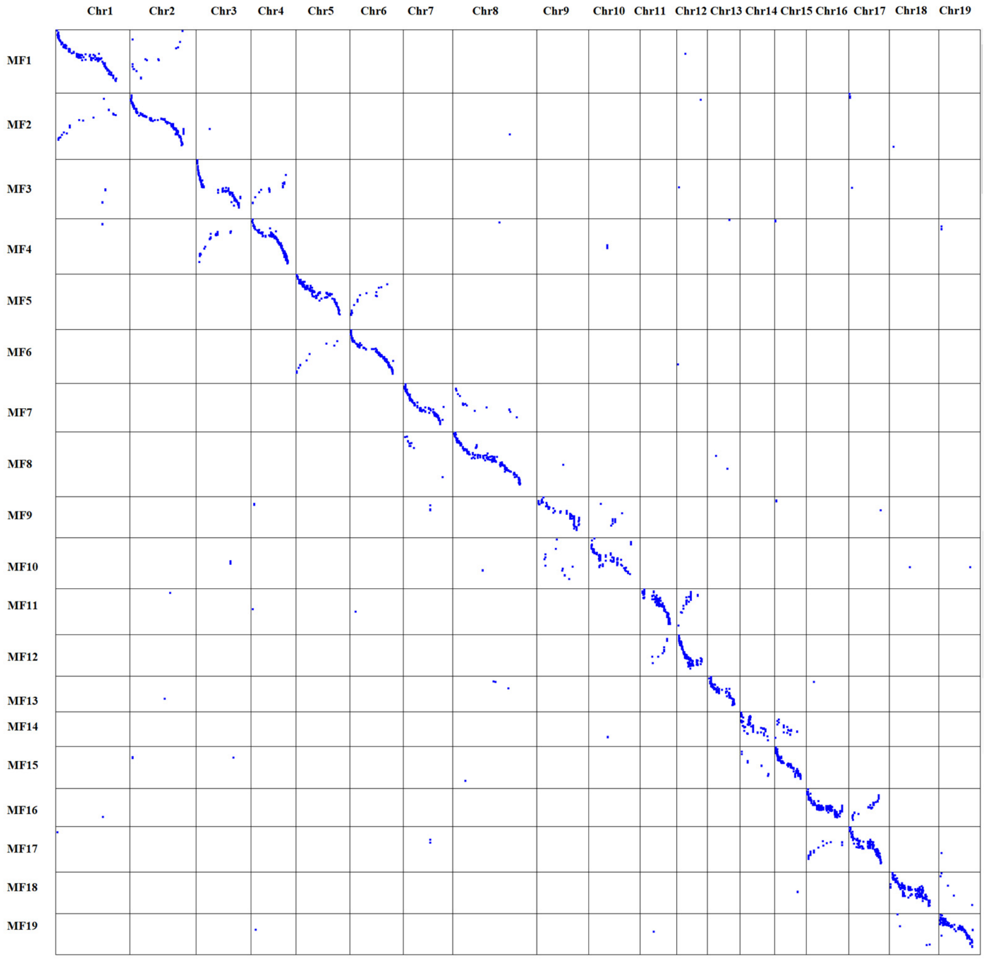


**Supplementary Figure 3. Comparison of SNP markers on the physical map (x axis) with their position on the genetic map (y axis).**

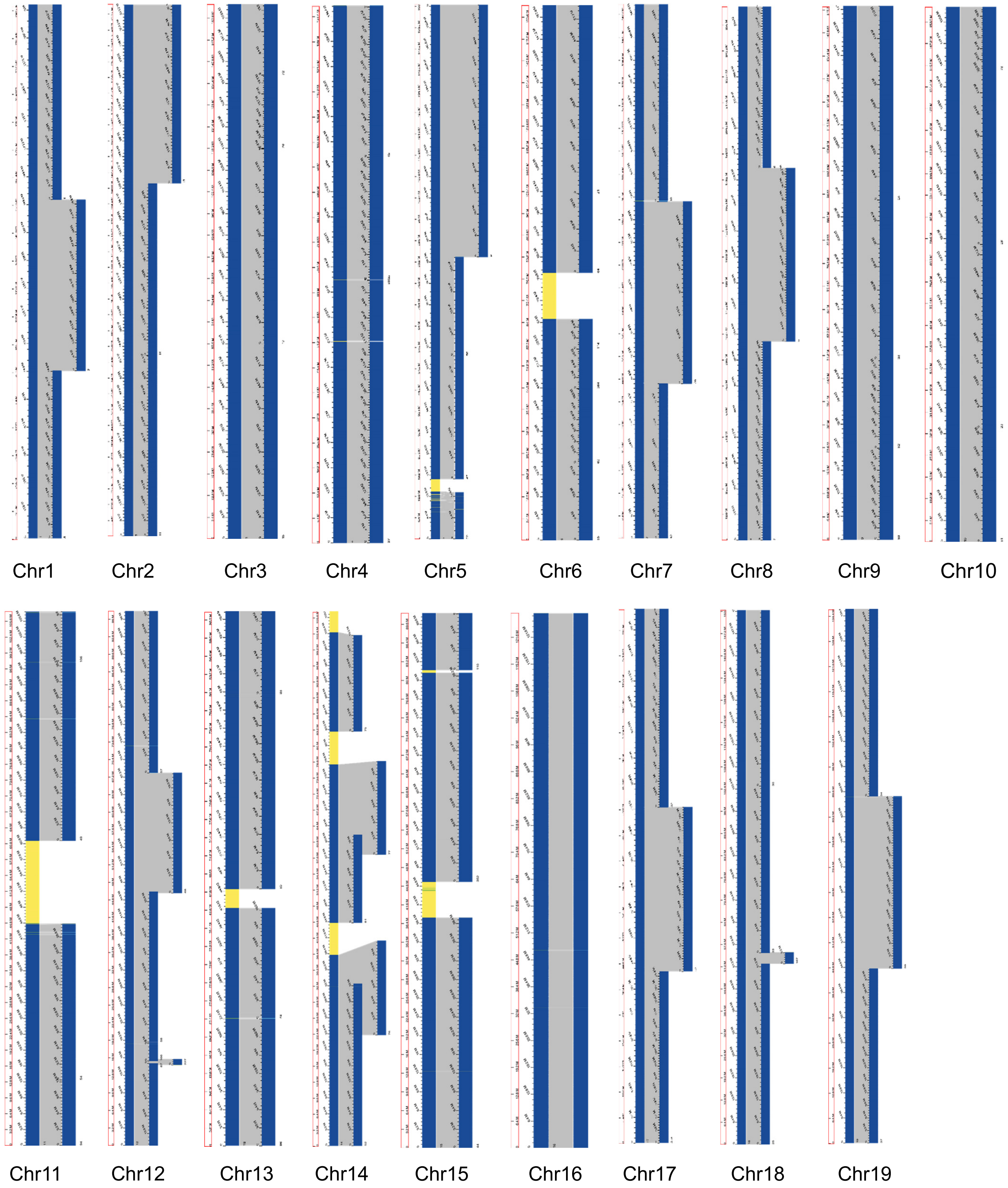


**Supplementary Figure 4. Bionano alignment images of *M. floridulus* assembly after manual correction.** The left blue lines of each chromosome represent the corrected Hi-C assembly results, the right blue lines of each chromosome represent the assembly scaffolds by optical mapping, and gray lines represent the collinear regions.

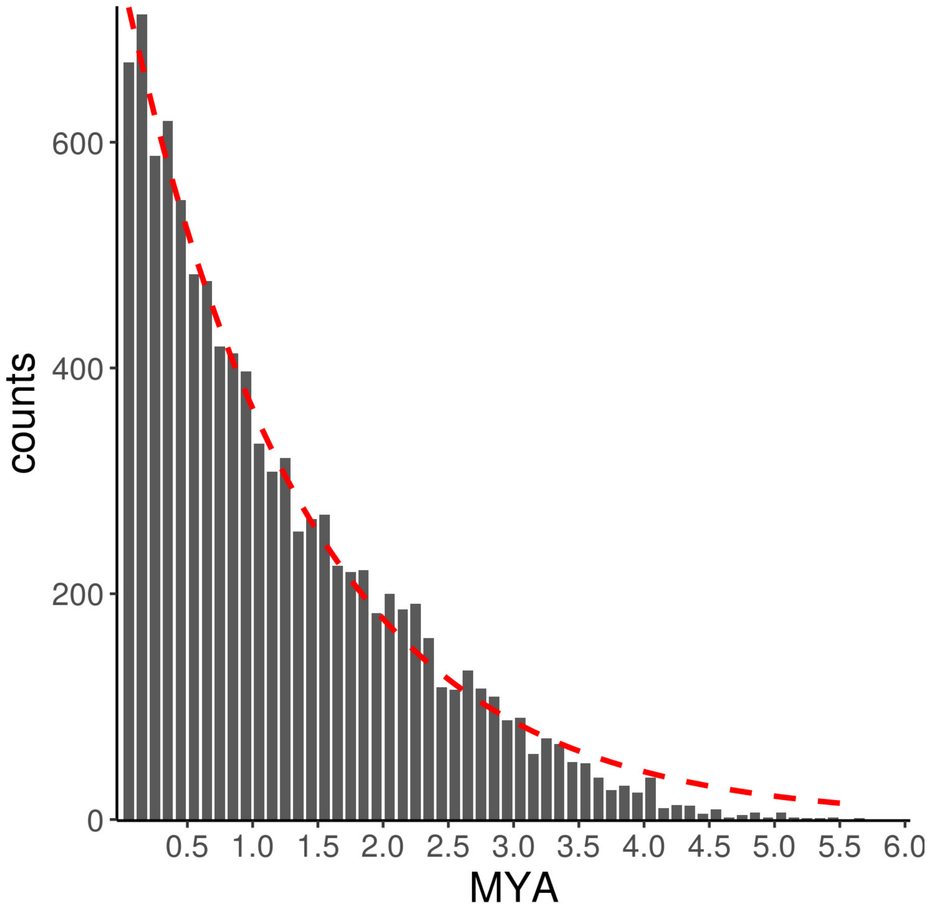


**Supplementary Figure 5. The distribution of insertion time of intact LTR retrotransposons in *M. floridulus*.** MYA, million years ago. y axis represents the number of LTR retrotransposon.

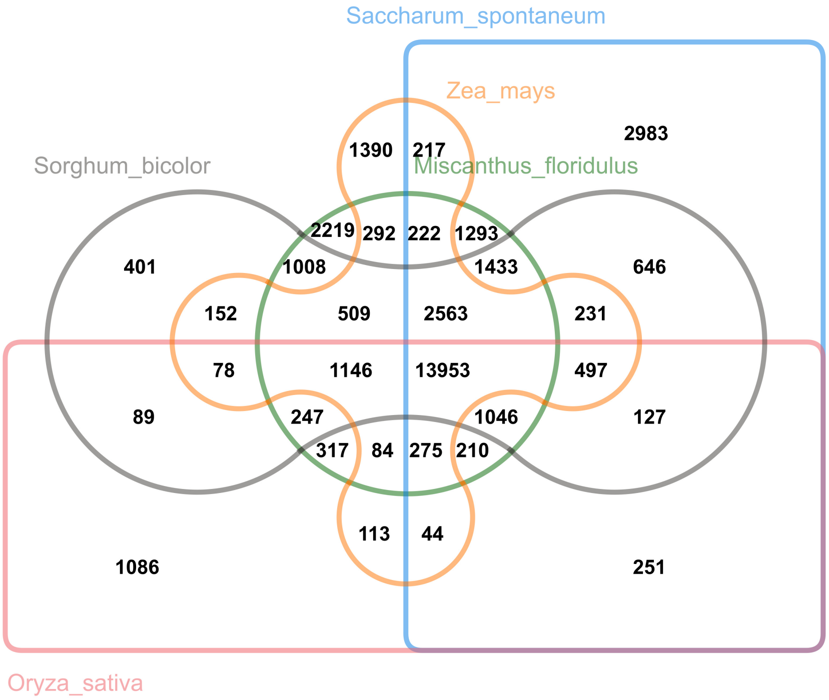


**Supplementary Figure 6. Orthologous gene families among *M. floridulus*, *S. spontaneum*, *S. bicolor*, *O. sativa* and *Z. mays*.** The numbers of gene families (clusters) are indicated for each species and species intersection. The total number of gene families in each species were 26,733 (*M. floridulus*), 22,275 (*Z. mays*), 25,991 (*S. spontaneum*), 24,126 (*S. bicolor*) and 19,563 (*O. sativa*), respectively.

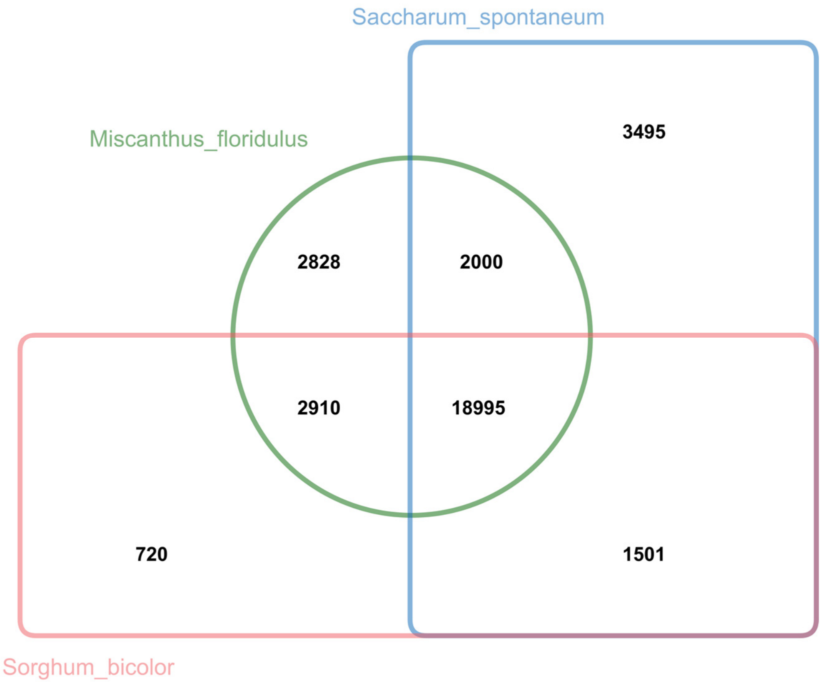


**Supplementary Figure 7. Orthologous gene families among *M. floridulus*, *S. spontaneum*, sorghum.** The numbers of gene families (clusters) are indicated for each species and species intersection

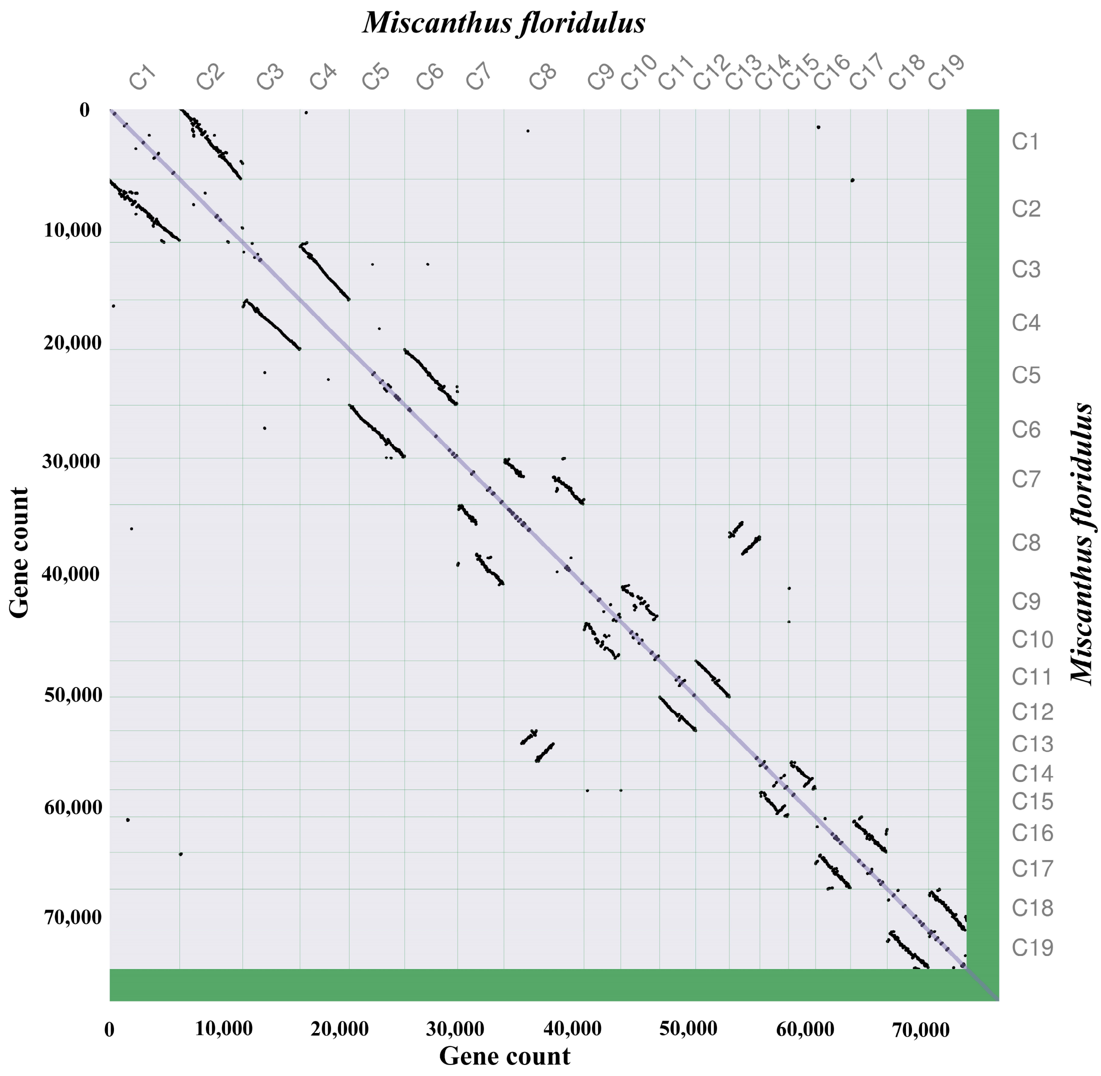


**Supplementary Figure 8. Alignment of *M. floridulus* chromosomes with itself**

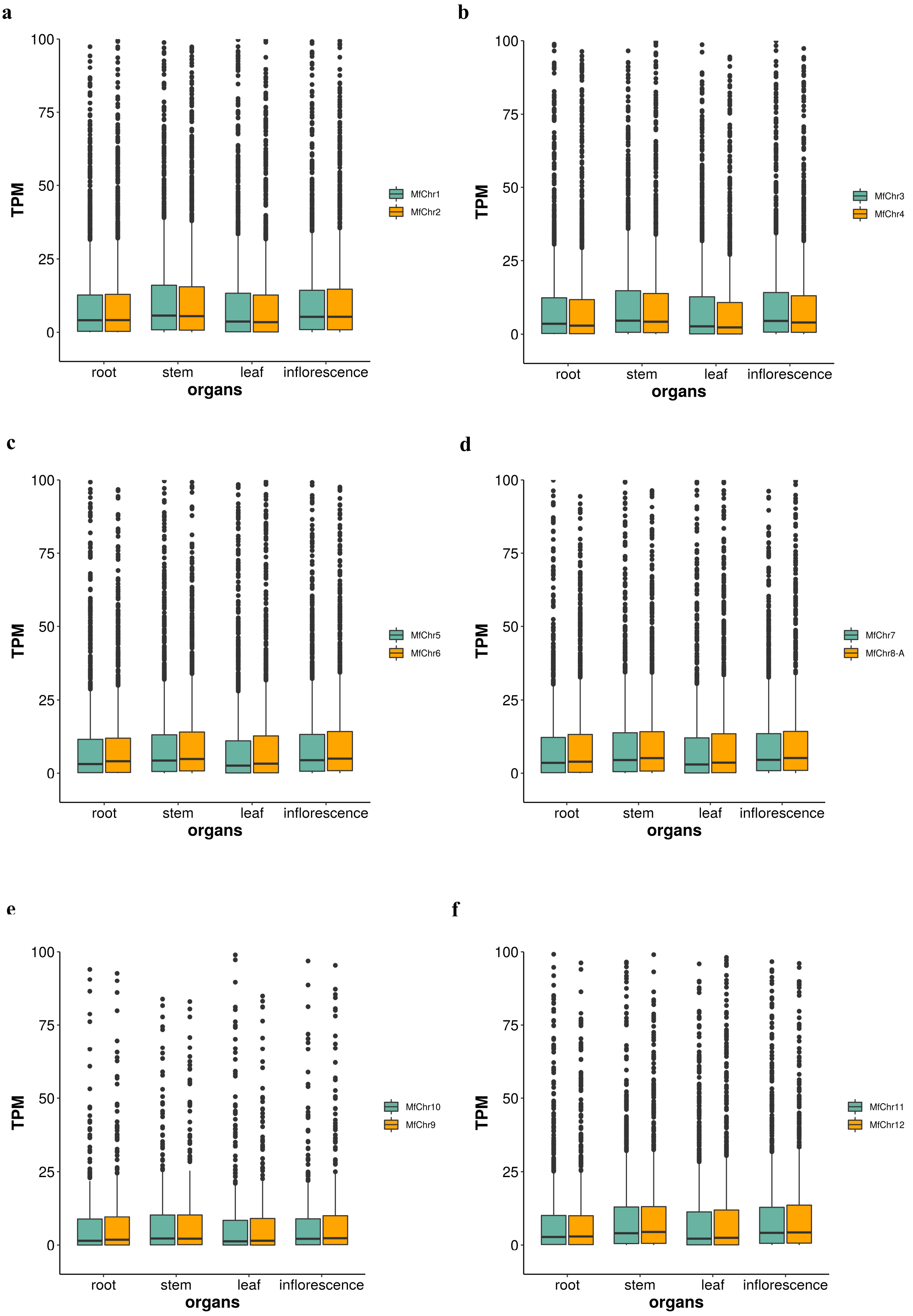
a
b
c
d
e
f
TPM
organs
root
stem
leaf
inflorescence
0
25
50
75
100
MfChr1
MfChr2
MfChr3
MfChr4
MfChr5
MfChr6
MfChr7
MfChr8-A
MfChr10
MfChr9
MfChr11
MfChr12

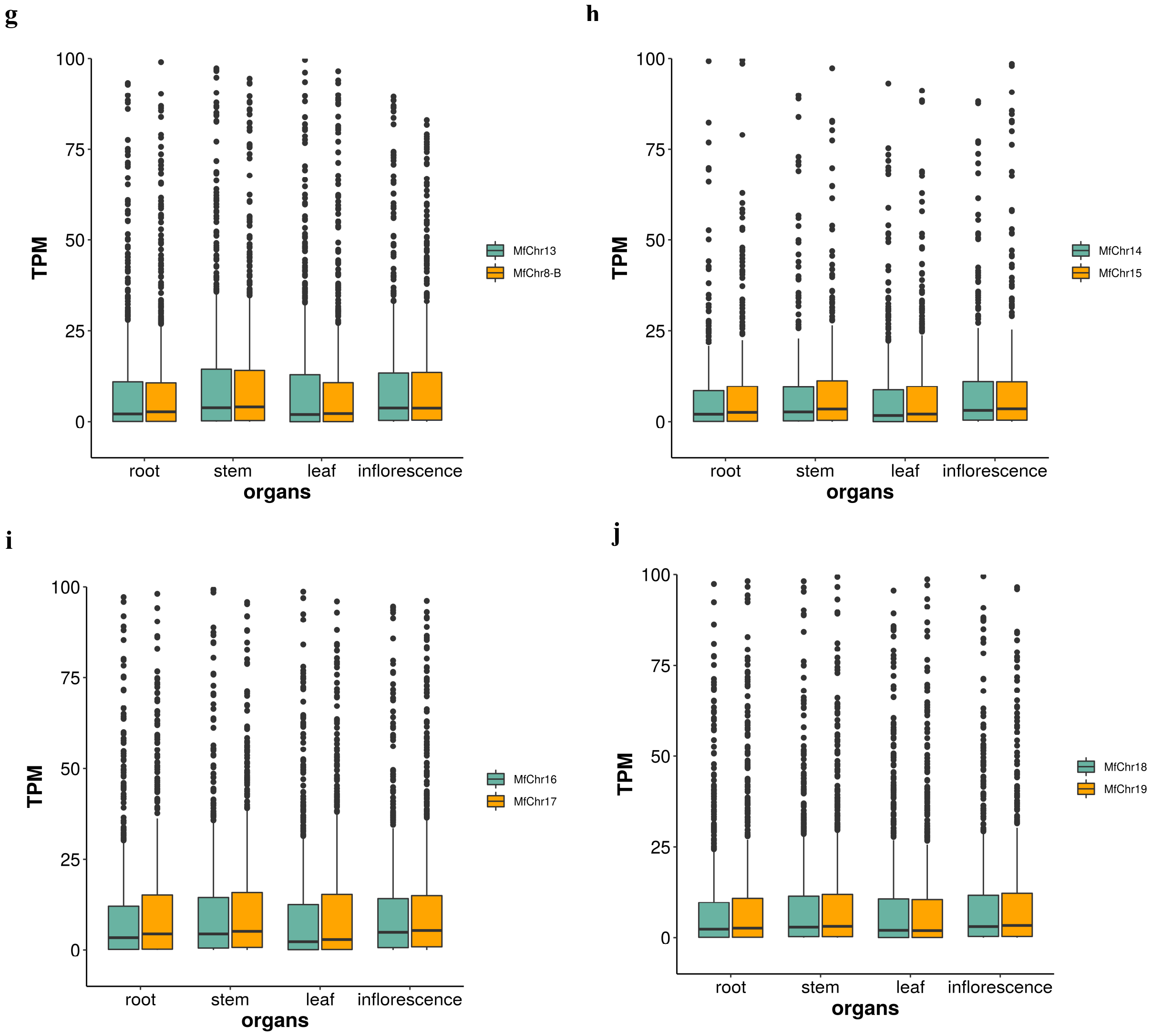


**Supplementary Figure 9. The gene expression of syntelogs on the subgenomes of *M. floridulus*.** Each figure shows that the average expression level of all syntelogs on the homoeologous chromosomes. In the boxplots, centre lines show the medians; box limits indicate the 25th and 75th percentiles as determined by the geom_boxplot function of the ggplot2 package from R; whiskers extend to TPM value equal to 100, individual data points are represented by dots. For the expression level of each gene, n=3 biologically independent samples for each tissue.

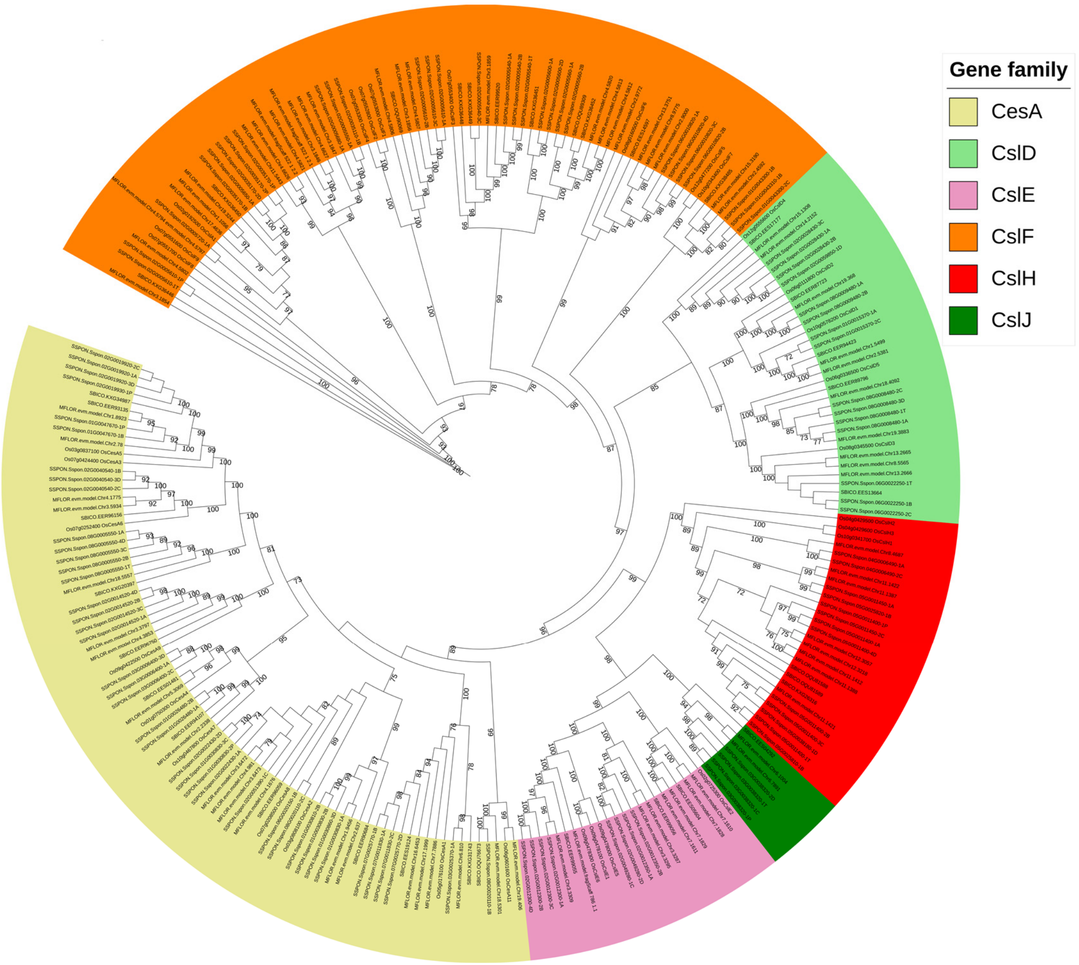


**Supplementary Figure 10. Phylogenetic tree of *CesA/CsI* genes from rice, sorghum, *M. floridulus* and *S. spontaneum*.** The different color ranges indicate different gene families.

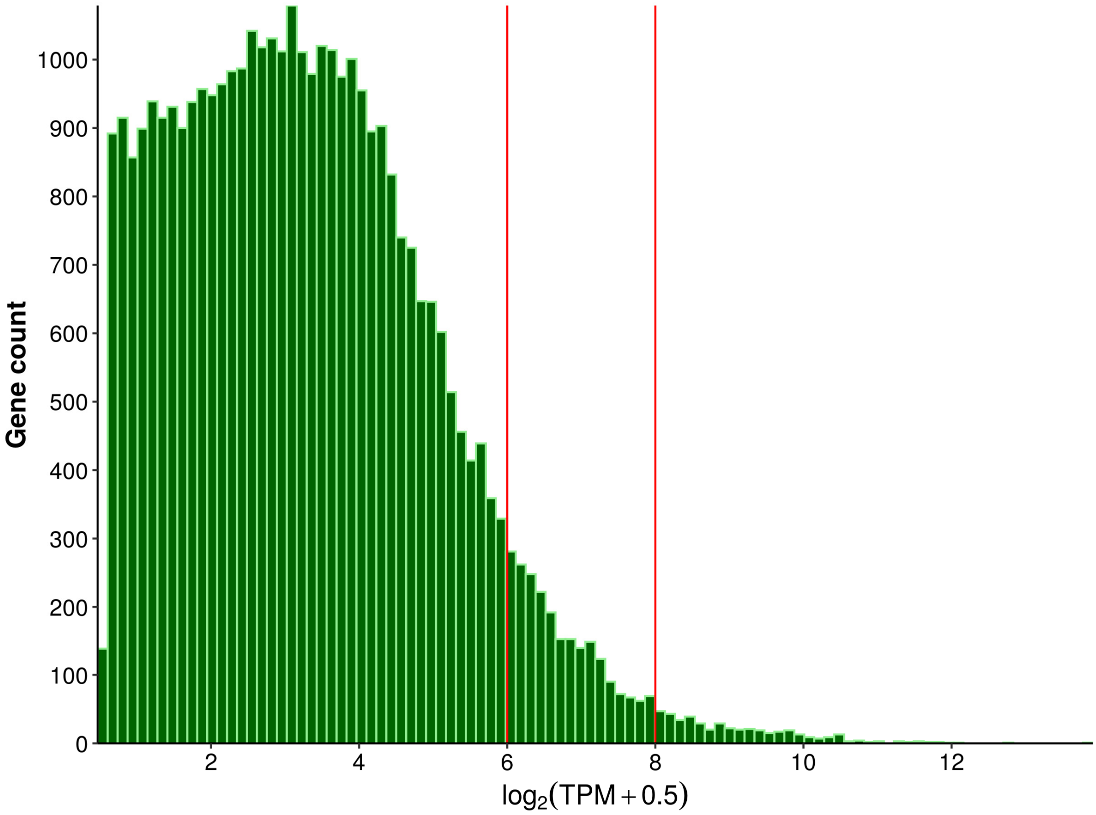


**Supplementary Figure 11. The histogram of genes with different expression level in growing stems.** The y-axis indicates the $\log_2$(TPM + 0.5). Most *CesA* and several *Csl* genes were highly expressed within the range marked by the two vertical red lines.

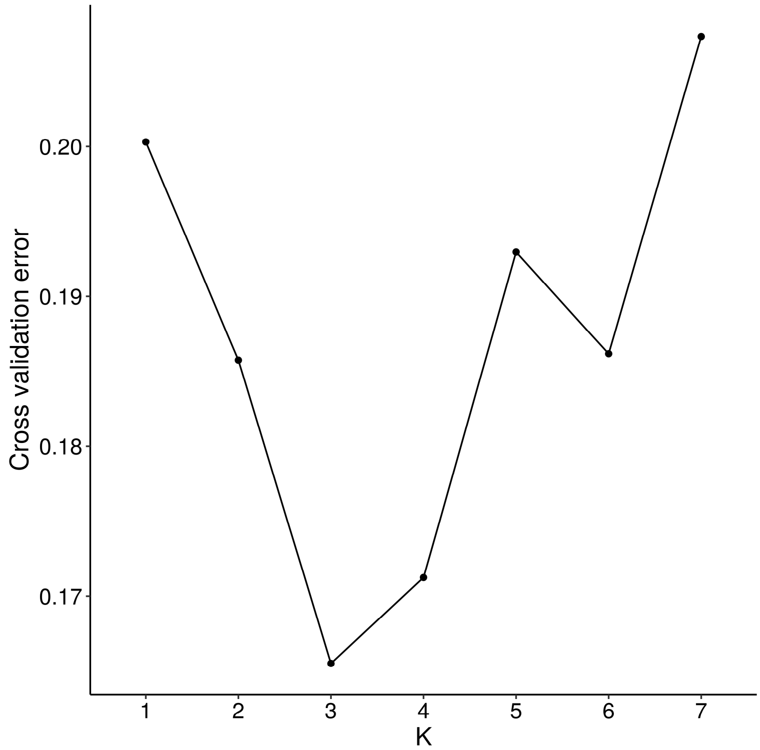


**Supplementary Figure 12. Cross validation errors show K=3 is the optimal population cluster grouping**

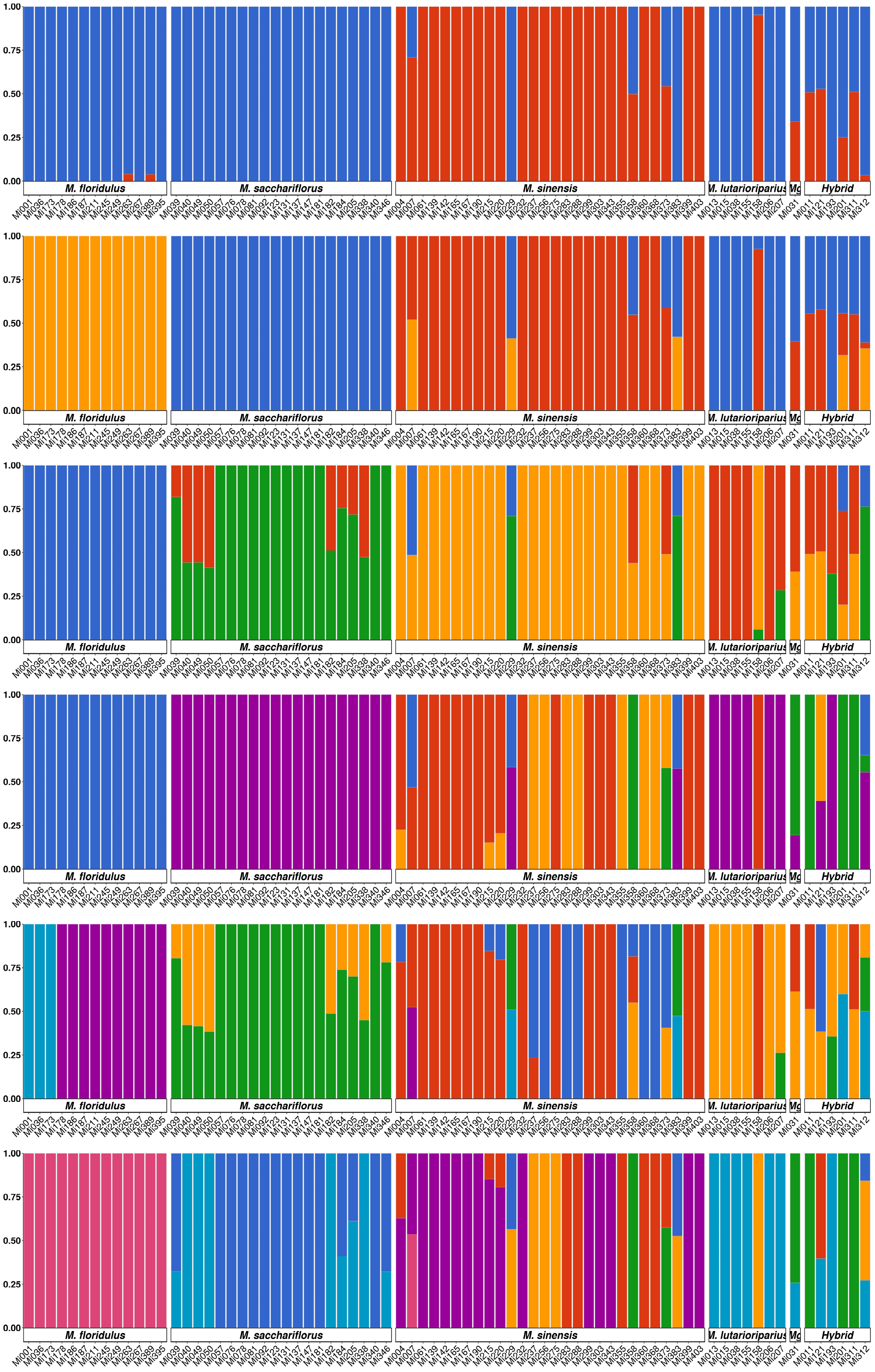


**Supplementary Figure 13. Population structure was showed with populations from K=2 to K=7 (from top to bottom)** Mg: *Miscanthus*×*giganteus*

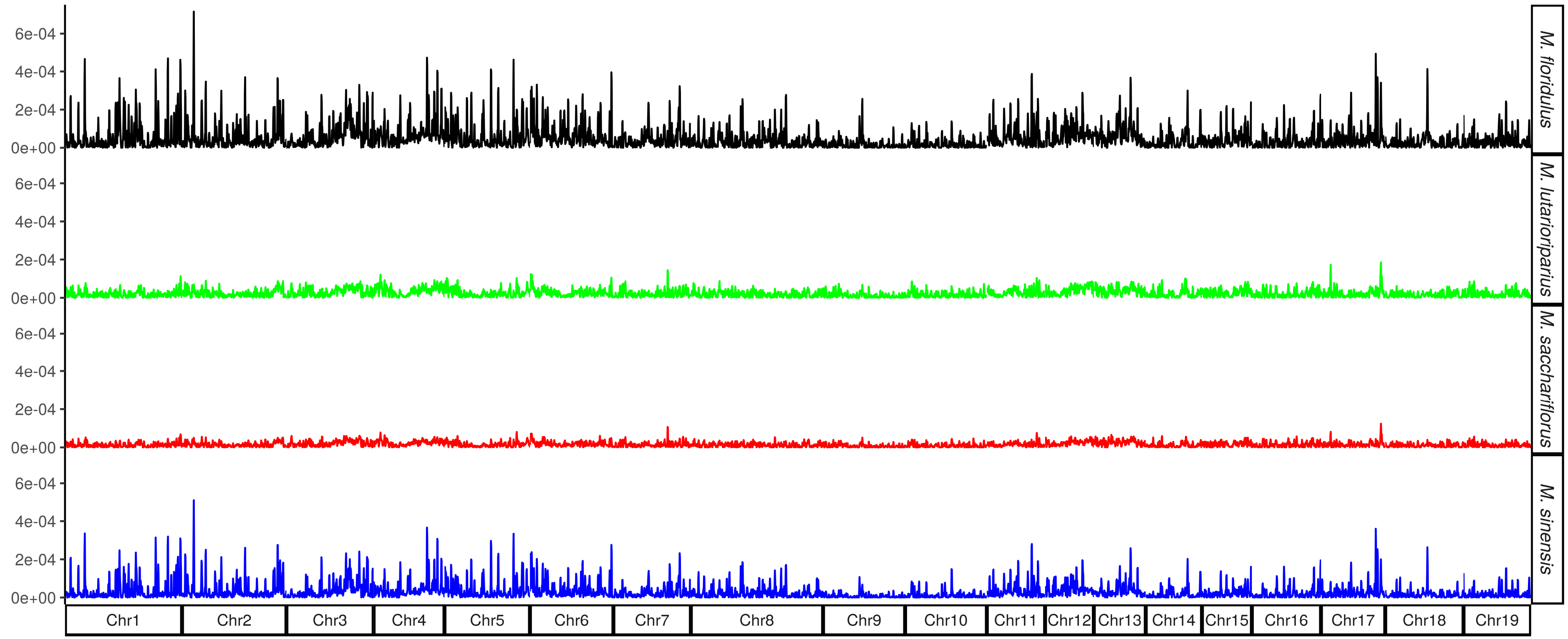


**Supplementary Figure 14. Nucleotide diversity (pi) for different species**

**Supplementary Note 1. Phased diploid genome assembly of *M. floridulus***

Due to the high heterozygosity nature of *M. floridulus* genome**,** FALCON-Unzip[1] and FALCON-Phase[2] software were employed to solve the problem of haplotype switching in diploid genome assemblies. By using the Pacbio long reads, FALCON generated "primary contigs" (p-contigs), which can be thought of as the longest continuous stretches of contiguously assembled sequence, and "alternative contigs" (a-contigs), which can be thought of mostly as structural variants that occur over the length of the primary contigs.

FALCON-Unzip algorithm used the heterozygosity information within the initial primary contigs for haplotype phasing and generated a subsequent set of p-contigs and the final haplotig sets (h-contigs) that represented more contiguous haplotype-specific sequence information than the a-contigs. Thus, p-contigs and h-contigs were obtained[1].

Hi-C, a method originally developed to study three-dimensional folding of the genome and physical interactions linking regulatory elements with distant sequences, is now used for generating chromosome-scale assemblies and haplotypes from short-read data[3]. FALCON-Phase is a new program that resolves phase-switches by reconstructing contig-length phase blocks using Hi-C short-reads mapped to both homozygous regions and phase blocks. Thus we used Hi-C data together with the p-contigs and h-contigs (output from FALCON-Unzip) as input for FALCON-Phase. The software then outputted two phased full-length pseudo-haplotypes for each primary contig[2]. One of the primary contig set was then integrated with 10X Genomics data to create a hybrid assembly for the final scaffolding and chromosome-scale assembly with Hi-C data.

**Supplementary Note 2. Analysis of RNA-Seq data**

For syntelog expression analysis and *CesA/Csl* genes expression analysis by RNA-Seq, mRNA was extracted from four different tissues of the same one mature *M. floridulus* plant at the beginning of heading. The *M. floridulus* plant was transplanted in April 2017 and grown in the greenhouse with temperatures at 32/28 °C in a 12 h light/12 h dark cycle. The samples were collected at 10:00-11:00 am, Aug 8, 2020 including primary root, growing stem (the first and second elongated internodes from the top), leaf (the third and fifth leaves from the top), and immature inflorescence. RNA extraction was performed with the NucleoSpin RNA Plant extraction kit (Machery-Nagel, Germany). The quality and quantity of RNA were monitored by a NanoDrop 2000c spectrophotometer (Thermo Scientifc, Wilmington, DE, USA) and agarose gel electrophoresis. mRNA was isolated from the total RNA by Dynabeads oligo (dT25) (Invitrogen Life Technologies, USA). RNA-seq libraries were constructed according to the TruSeq RNA Sample Prep manufacturer's instructions (Illumina Inc., San Diego, CA, USA). The normalized libraries with individual index were loaded onto Illumina HiSeq 2500 platform for cluster generation and sequencing. The data from three biological replicates were obtained by paired-end reads.

The RNA-seq reads were quality filtered by fastp[4] and then mapped to *M. floridulus* cDNA sequences by Salmon[5] with the default parameters. Meanwhile, TPM values were also calculated by Salmon. For syntelog expression analysis, the expression of syntelogs across different tissues in each of homoeologous chromosome were retrieved and plotted via the R software. The expression of *CesA/Csl* genes were retrieved and heatmap was drawn using the pheatmap package (https://CRAN.R-project.org/package=pheatmap) of R software with values

of $\log_2(\text{TPM} + 0.5)$ .

## Supplementary Note 3. Phylogenetic tree of CesA/Csl family

At first, the *CesA/Csl* genes were retrieved from the files generated from the Interproscan-based functional annotation with Pfam accession ID “PF03552”. PREQUAL[6] was used to identify and mask regions with non-homologous adjacent characters in FASTA files of CesA/Csl protein sequences. The prefiltered CesA/Csl protein sequences were then aligned by PRANK[7], probabilistic multiple alignment program for amino-acid sequences which is based on a novel algorithm that treats insertions correctly and avoids over-estimation of the number of deletion events. IQ-TREE[8], a fast and effective stochastic algorithm for estimating maximum-likelihood phylogenies, were then employed to construct the phylogenetic tree of aligned CesA/Csl proteins. We called this pipeline as “PREQUAL-PRANK-IQ-TREE” pipeline.

As the CesA phylogenetic tree was constructed, we found that there was a new group, which was not homologous to any rice CesA proteins and was not clustered with any other groups. We then used one member of this family as query against the non-redundant proteins database in NCBI via BLASTP and the resulting proteins with high score was mostly members of CesA5 family from both monocots and dicots.

We thus used other online pipelines of NGPhylogeny.fr[9] website, which provides different combinations of multiple alignment programs, alignment curation programs and tree inference methods. Several pipelines were tested as follows:

(1). MAFFT[10] or MUSCLE[11] (multiple alignment) + BMGE[12] or Gblocks[13] (alignment curation) + PhyML[14] (tree inference based on maximum likelihood method)

(2). MAFFT or MUSCLE (multiple alignment) + BMGE or Gblocks (alignment curation) + MrBayes[15] (tree inference based on Bayesian method)

However, no matter which pipeline was used, we consistently got the same results that members of this family did not group with CesA5 or other clades, which suggested the existence of this new family in Saccharinae. Thus, we renamed the family as CesAX. Please note that the genes of this family in *M. floridulus* were low expressed in Supplementary Data 3.

## Supplementary Note 4. Analysis of resequencing data of different accessions from the genus *Miscanthus*

Because many accessions of *Miscanthus* for resequencing don’t belong to the species *M. floridulus*, uniquely-mapped reads were extracted from the unfiltered BAM files, which were generated by BWA-MEM[16] mapping. The criteria for extraction was highly strict with Sambamba[17] parameters “-F "mapping_quality” >= 30 and not (unmapped or secondary_alignment) and not ([XA] != null or [SA] != null)”. Then the extracted uniquely-mapped reads were used for subsequent variant calling.

The statistics of uniquely-mapped reads were supplied in Supplmental Data 5. Please note the calculation of depth of mapped reads. The standard formula for its calculation is “Accumulated depth of sites covered by mapped reads / Total sites of genome”. Howerver, the depth would be

relatively low if only a small set of sites covered by high-quality mapped reads were selected for calculation, just like the exome-sequencing. Thus, new formula “Accumulated depth of sites covered by mapped reads / Total sites covered by mapped reads” was applied to calculate the average depth of sites covered by uniquely-mapped reads. The result was added in the last column of Supplementary Data 5.

The variants were then filtered by a custom script to change low quality data (GQ < 30 or DP < 4 or DP >50) to null and further filtered by SnpSift[18] with the parameters "QUAL > 30 & NS >= 63 & (QUAL / AO > 10) & SAF > 0 & SAR > 0 & RPR > 1 & RPL > 1". Afterwards, the multi-allelic genotype of polyploids were filtered and converted to bi-allelic genotype by our custom script. Finally, the remaining variants were used for downstream analysis.

**References**


1. Chin, C.-S. et al. Phased diploid genome assembly with single-molecule real-time sequencing. *Nat. Methods* **13**, 1050 (2016).
2. Kronenberg, Z.N. et al. Extended haplotype phasing of de novo genome assemblies with FALCON-Phase. *bioRxiv*, 327064 (2019).
3. Korbel, J., Lee, C. Genome assembly and haplotyping with Hi-C. *Nat. Biotechnol*. **31**, 1099–1101 (2013).
4. Chen, S., Zhou, Y., Chen, Y. & Gu, J. fastp: an ultra-fast all-in-one FASTQ preprocessor. *Bioinformatics* **34**, i884-i890 (2018).
5. Patro, R., Duggal, G., Love, M.I., Irizarry, R.A. & Kingsford, C. Salmon provides fast and bias-aware quantification of transcript expression. *Nat. Methods* **14**, 417 (2017).
6. Whelan, S., Irisarri, I., & Burki, F. PREQUAL: detecting non-homologous characters in sets of unaligned homologous sequences. *Bioinformatics* **34**, 3929 (2018).
7. Löytynoja, A., & Goldman, N. webPRANK: a phylogeny-aware multiple sequence aligner with interactive alignment browser. *BMC Bioinformatics* **11**, 579 (2010).
8. Nguyen, L. T., Schmidt, H. A., Haeseler, A. V., & Minh, B. Q. IQ-TREE: A fast and effective stochastic algorithm for estimating maximum likelihood phylogenies. *Mol. Biol. Evol.* **32**, 268 (2015).
9. Lemoine, F., Correia, D., Lefort, V., Doppelt-Azeroual, O., Mareuil, F., Cohen-Boulakia, S., & Gascuel, O. NGPhylogeny.fr: new generation phylogenetic services for non-specialists. *Nucleic Acids Res.* **47**, W250 (2019).
10. Katoh, K., Misawa, K., Kuma, K., & Miyata, T. MAFFT: a novel method for rapid multiple sequence alignment based on fast Fourier transform. *Nucleic Acids Res*. **30**, 3059 (2002).
11. Edgar, R.C. MUSCLE: a multiple sequence alignment method with reduced time and space complexity. *BMC Bioinformatics* **5**, 113 (2004).
12. Criscuolo, A., Gribaldo, S. BMGE (Block Mapping and Gathering with Entropy): a new software for selection of phylogenetic informative regions from multiple sequence alignments. *BMC Evol. Biol*. **10**, 210 (2010).
13. Castresana, J. Selection of conserved blocks from multiple alignments for their use in phylogenetic analysis. *Mol. Biol. Evol.* **17**, 540 (2000).
14. Guindon, S., Delsuc, F., Dufayard, J.F., Gascuel, O. Estimating maximum likelihood phylogenies with PhyML. *Methods Mol. Biol.* **537**, 113 (2009).
15. Huelsenbeck, J. P., & Ronquist, F. MRBAYES: Bayesian inference of phylogenetic trees.

*Bioinformatics* **17**, 754 (2001).

16. Li, H. Aligning sequence reads, clone sequences and assembly contigs with BWA-MEM. arXiv preprint *arXiv*:**1303** 3997 (2013).
17. Tarasov, A., Vilella, A.J., Cuppen, E., Nijman, I.J. & Prins, P. Sambamba: fast processing of NGS alignment formats. *Bioinformatics* **31**, 2032-2034 (2015).
18. Ruden, D.M. et al. Using Drosophila melanogaster as a model for genotoxic chemical mutational studies with a new program, SnpSift. *Front. Genet.* **3**, 35 (2012).
